# Supplementary material for: A pragmatic group sequential, placebo-controlled, randomised trial to determine the effectiveness of glyceryl trinitrate for retained placenta (GOT-IT): a study protocol
Source: BMJ Open. 2017 Sep 18;7(9):e017134. doi: 10.1136/bmjopen-2017-017134 (PMC5623532; doi:10.1136/bmjopen-2017-017134)
Supplement: Author's manuscript [file bmjopen-2017-017134.draft_revisions.pdf]

BMJ Open

BMJ Open is committed to open peer review. As part of this commitment we make the peer review history of every article we publish publicly available.

When an article is published we post the peer reviewers' comments and the authors' responses online. We also post the versions of the paper that were used during peer review. These are the versions that the peer review comments apply to.

The versions of the paper that follow are the versions that were submitted during the peer review process. They are not the versions of record or the final published versions. They should not be cited or distributed as the published version of this manuscript.

BMJ Open is an open access journal and the full, final, typeset and author-corrected version of record of the manuscript is available on our site with no access controls, subscription charges or pay-per-view fees (http://bmjopen.bmj.com).

If you have any questions on BMJ Open's open peer review process please email editorial.bmjopen@bmj.com

GOT-IT: A pragmatic group sequential placebo controlled randomised trial to determine the effectiveness of Glyceryl trinitrate for retained placenta: a study protocol

Journal:	*BMJ Open*
Manuscript ID	bmjopen-2017-017134
Article Type:	Protocol
Date Submitted by the Author:	03-Apr-2017
Complete List of Authors:	Denison, Fiona; The University of Edinburgh , MRC Centre for Reproductive Health<br>Norrie, John; Edinburgh Clinical Trials Unit, University of Edinburgh No. 9, Bioquarter<br>Lawton, Julia; University of Edinburgh, Centre for Population Health Sciences<br>Norman, Jane; University of Edinburgh, MRC Centre for Reproductive Health<br>Scotland, Graham; University of Aberdeen, Health Economics Research Unit<br>McPherson, Gladys; Health Services Research Unit Centre for Healthcare Randomised Trials Health Services Research Unit<br>McDonald, Alison; University of Aberdeen, The Centre for Healthcare Randomised Trials (CHaRT), Health Services Research Unit, Foresterhill<br>Forrest, Mark; The Centre for Healthcare Randomised Trials (CHaRT), Health Services Research Unit, University of Aberdeen, Foresterhill<br>Hudson, Jemma; The Centre for Healthcare Randomised Trials (CHaRT), Health Services Research Unit, University of Aberdeen, Foresterhill<br>Brewin, Jane; Tommy's,<br>Peace, Mathilde<br>Clarkson, Cynthia<br>Brook-Smith, Sheonagh; NHS Lothian, Simpson’s Centre for Reproductive Health, Royal Infirmary of Edinburgh<br>Morrow, Susan; The University of Edinburgh, Asthma UK Centre for Applied Research, Centre for Medical Informatics, Usher Institute of Population Health Sciences and Informatics<br>Hallowell, Nina; Ethox Centre, Nuffield Department of Population Health, University of Oxford, Old Road<br>Hodges, Laura; University of Edinburgh, MRC Centre for Reproductive Health Edinburgh<br>Carruthers, Kathryn; University of Edinburgh, Centre for Cardiovascular Science
**Primary Subject Heading**:	Obstetrics and gynaecology
Secondary Subject Heading:	Obstetrics and gynaecology, Health economics, Evidence based practice
Keywords:	retained placenta, randomised controlled trial, Glyceryl trinitrate

For peer review only - http://bmjopen.bmj.com/site/about/guidelines.xhtml

For peer review only

GOT-IT: A pragmatic group sequential placebo controlled randomised trial to determine the effectiveness of Glyceryl trinitrate for retained placenta: a study protocol

Denison FC[1]*, Norrie J[2], Lawton J[3], Norman JE[1], Scotland G[4], McPherson G[2], McDonald A[2], Forrest M[2], Hudson J[2], Brewin J[5], Peace M[1], Clarkson C[1], Brook-Smith S[6], Morrow S[7], Hallowell N[3,8], Hodges L[1], Carruthers KF[1]

* Corresponding author:

Fiona Denison: Fiona.Denison@ed.ac.uk

[1]Tommy's Centre for Maternal and Fetal Health Research, Medical Research Council Centre for Reproductive Health, Queen's Medical Research Institute, University of Edinburgh, 47 Little France Crescent, Edinburgh, UK

[2]The Centre for Healthcare Randomised Trials (CHaRT), Health Services Research Unit, University of Aberdeen, Foresterhill, Aberdeen, UK

[3]Centre for Population Health Sciences, University of Edinburgh, Edinburgh, UK

[4]Health Economics Research Unit, Institute of Applied Health Sciences, University of Aberdeen, Foresterhill, Aberdeen, UK

[5]Tommy's, Nicholas House, 3 Laurence Poutney Hill, London, UK

[6]Simpson's Centre for Reproductive Health, Royal Infirmary of Edinburgh, Edinburgh, UK

[7]Usher Institute of Population Health Sciences and Informatics, University of Edinburgh

[8]Ethox Centre, Nuffield Department of Population Health, University of Oxford, UK

For peer review only

ABSTRACT:

Introduction

A retained placenta is diagnosed when the placenta is not delivered following delivery of the baby. It is a major cause of postpartum haemorrhage and treated by the operative procedure of manual removal of placenta. The aim of this pragmatic randomised placebo controlled double blind UK-wide trial, with an internal pilot to provide reassurance on all the trial processes and nested qualitative research to adjust strategies to refine delivery of the main trial, is to determine whether sublingual GTN is (or is not) clinically and cost effective for (medical) management of retained placenta.

Methods and analysis

The primary clinical outcome is need for manual removal of placenta, defined as the placenta remaining undelivered 15 minutes post study treatment and/or being required within 15 minutes of treatment due to safety concerns. The primary safety outcome is measured blood loss between administration of treatment and transfer to the postnatal ward or other clinical area. The primary patient-sided outcome is satisfaction with treatment and a side effect profile. The primary economic outcome is net incremental costs (or cost savings) to the National Health Service of using GTN versus standard practice. Secondary outcomes are being measured over a range of clinical and economic domains. Health service costs will be compared with multiple trial outcomes in a cost-consequence analysis of GTN versus standard practice.

Ethics and dissemination

Ethical approval has been obtained from the North East- Newcastle & North Tyneside 2 Research Ethics Committee [13/NE/0339]. Dissemination plans for the trial include the Health Technology Assessment Monograph, presentation at international scientific meetings, and publication in high impact peer reviewed journals.

Trial Registration

For peer review only

For peer review only - http://bmjopen.bmj.com/site/about/guidelines.xhtml

The trial is funded by the HTA (12/29/01) and has been registered on the International Standard Randomised Controlled Trial Number (ISRCTN) as ISCRTN88609453.

For peer review only

For peer review only - http://bmjopen.bmj.com/site/about/guidelines.xhtml

Strengths and limitations of this study

- Pragmatic randomised placebo controlled double blind UK-wide trial to determine whether sublingual GTN is (or is not) clinically and cost effective for (medical) management of retained placenta.
- Inclusion of clinical, patient-sided, safety and economic outcomes with minimal exclusion criteria.
- Pragmatic approach to obtaining consent and GCP-training to facilitate recruitment in the emergency setting
- Fully adaptive group sequential design
- Inability to differentiate whether a placenta is adherent or trapped prior to administration of study drug

For peer review only

For peer review only - http://bmjopen.bmj.com/site/about/guidelines.xhtml

1.0 INTRODUCTION

1.1 Background

A retained placenta is diagnosed when the placenta is not delivered within 30 or 60 minutes after delivery of the baby following active or physiological management of the third stage of labour, respectively [1] and is a major cause of postpartum haemorrhage [2, 3]. The incidence of retained placenta is rising in the UK due to changes in maternal demographics, increased intrapartum interventions and operations involving exploration of the uterine cavity including surgical termination of pregnancy and dilation and curettage [4]. Retained placenta currently affects approximately 2% of vaginal deliveries [4] which equates to nearly 11,000 women per annum in the UK. Following failure of active or physiological management, the National Institute of Health and Care Excellence recommends that retained placenta should be treated by the operative procedure of manual removal of the placenta [1]. This procedure requires skilled personnel, an operative environment and, compared to spontaneous placental delivery, has its own attendant risks, including bleeding [3] and infection [5]. There is often a delay in undertaking the operation, which leads to further haemorrhage [6], and the procedure itself is costly in terms of staff time. Furthermore, the infrastructure required for this operative intervention is not available in all delivery settings and the invasive nature of this procedure has the potential to delay or interrupt mother-baby bonding in the immediate post-partum period. To reduce the number of women requiring manual removal of placenta, there is therefore a need for new (and effective) medical treatments for retained placenta. The reduction in operative interventions would potentially have cost benefits for the National Health Service (NHS) and also for women in terms of increased satisfaction, less separation between mother and baby immediately after birth, and reduced morbidity.

1.2 Rationale for study

Observational studies have suggested that nitric oxide donors including glyceryl trinitrate (GTN) given intravenously, might be effective for the management of retained placenta with success rates

For peer review only - http://bmjopen.bmj.com/site/about/guidelines.xhtml

ranging between 94% to 100% [7-10]. Although intravenous GTN appears to be efficacious for retained placenta in these small studies, this route of administration is not practical in all settings and intravenous administration causes unacceptable side-effects including symptomatic hypotension at higher doses [3]. More recently, sublingual administration has been trialed, with studies reporting experience with sublingual GTN tablet and spray. Using a sublingual GTN tablet, two small studies suggest a beneficial effect of sublingual GTN tablet; however a third larger study does not (RCT ISRCTN34755982; 37.3% vs 20.4%; GTN vs placebo; $p > 0.05$; n=105 women) [11]. The alternative preparation for sublingual administration is GTN spray [12]. Compared to the tablet preparation, sublingual GTN spray has several advantages including stability at room temperature, significant reduction in latency of onset with onset beginning at 30-45 seconds, peaking at 90-120 seconds and lasting up to five minutes [13-15], and fewer objective and subjective side-effects [16]. GTN spray is accepted obstetric management in other scenarios where rapid tocolysis is required such as uterine relaxation for release of a trapped head in breech delivery or at caesarean section [12]. Furthermore, anecdotal reports suggest that GTN spray may also have a use in management of retained placenta [12].

To summarise, although a growing body of evidence supports GTN use for the treatment of retained placenta, much of this evidence is based on anecdotal case-reports or clinical studies which are non-randomised, do not included a placebo arm and are underpowered. We are therefore currently undertaking a randomised placebo controlled double blind pragmatic group sequential UK-wide (GOT-IT trial) to definitively determine whether sublingual GTN is (or is not) clinically and cost effective for management of retained placenta.

1.3 Study objectives

The aim of this placebo controlled, double blind, pragmatic group sequential UK-wide trial is to determine the effectiveness and cost-effectiveness of GTN for treating retained placenta and avoiding manual removal of placenta.

For peer review only - http://bmjopen.bmj.com/site/about/guidelines.xhtml

The hypothesis being tested is that sublingual GTN spray is clinically and cost effective in treating retained placenta after vaginal delivery, reducing the need for manual removal of placenta following failure of current management.

The primary objectives are to: 1) determine the clinical effectiveness of sublingual GTN in treating retained placenta and avoiding manual removal of placenta in women with vaginal delivery following failure of current management (clinical domain), 2) determine the side-effect profile for GTN given to treat retained placenta (safety domain), 3) assess patient satisfaction with GTN given for retained placenta (patient-sided domain) and 4) assess the net costs (or cost savings) to the National Health Service of using GTN for the treatment of retained placenta compared to standard practice (economic domain)

The secondary objectives are to assess NHS costs in relation to the primary outcome and range of secondary outcomes which are expected to differ between the two arms of the trial, using a cost-consequence balance sheet approach.

The trial contained an internal pilot, which commenced in October 2014 and was completed in April 2015. The aim of this internal pilot, which recruited from eight sites across the UK, was to provide reassurance on all the trial processes, including recruitment, consent, randomisation, delivery of treatment, and follow-up assessments to ensure that all were running smoothly. Recruitment to the substantive trial is ongoing and is due to finish in September 2017.

A nested qualitative study was undertaken during the internal pilot to adjust strategies to (a) maximise recruitment into the main trial; (b) refine the recruitment and consent pathway and (c) ensure staff are given appropriate training and support to help promote the successful delivery of the main trial. In-depth qualitative interviews were undertaken with staff (n=27) involved in trial recruitment, consenting and/or trial delivery and with trial recruits (n=22) within, when possible, four weeks of randomisation. The results of the qualitative research have been published and have informed the delivery of the main trial [17, 18].

For peer review only - http://bmjopen.bmj.com/site/about/guidelines.xhtml

2.0 METHODS AND ANALYSIS

2.1 Study Design

The aim of this placebo controlled, double blind, pragmatic group sequential UK-wide trial is to determine the effectiveness of GTN for treating retained placenta and avoiding manual removal of placenta. Women are identified as being potentially eligible for the trial if, following vaginal birth, they have a retained placenta and are at risk of needing a manual removal of placenta after failure of current management of the third stage of labour. If eligible for the trial, women are treated with either the study drug or a placebo in a double-blind fashion. Following administration of the study medication, if the placenta still remains undelivered after 15 minutes, the treating clinician determines ongoing clinical management as per standard care.

The pre-specified group sequential monitoring plan requires the Data Monitoring Committee to look at accumulating unblinded data on five occasions. The Data Monitoring Committee is using the results of these analyses to advise the Trial Steering Committee on adapting the trial design to either a) stop prematurely for futility (no prospect of establishing a treatment effect of at least 10%), or b) stop prematurely if proof beyond a reasonable doubt is established that there is a convincing treatment benefit of at least 10%. Assuming the trial recruits to its maximum size, we are aiming to recruit 1100 participants (maximum number randomised 1086, with the uplift to 1100 to account for a small allowance for withdrawal of consent or other rare loss to follow-up) over 42 months.

2.2 Study Population

2.2.1 Setting

We are recruiting women from delivery wards in UK maternity hospitals. The delivery wards are of varying size and location ensuring the results of the trial will be generalisable to the UK. At present, recruitment is being undertaken in 28 sites across the UK.

For peer review only - http://bmjopen.bmj.com/site/about/guidelines.xhtml

2.2.2 Selection of participants

As is standard practice, clinicians are undertaking the initial eligibility screening. Women are identified as being potentially eligible for the trial if, following vaginal birth, they have a retained placenta and are at risk of needing a manual removal of their placenta after failure of current management of the third stage of labour, defined as the placenta remaining undelivered after (i) 30 minutes of active management or (ii) 60 minutes of physiological management plus a further 30 minutes of active management of the third stage of labour.

2.2.3 Planned inclusion and exclusion criteria

Inclusion criteria:

Women are eligible for inclusion if they are:

- Aged ≥16 years
- Vaginal delivery (including women with a previous caesarean section and an instrumental vaginal delivery in the delivery room)
- >14 weeks gestation
- Haemodynamically stable, defined as having a heart rate ≤119 beats per minute and a systolic blood pressure of > 100mmHg

Exclusion Criteria:

Women are excluded if they have one or more of the following:

- Unable to give informed consent
- Suspected placenta accreta/increta/percreta
- Multiple pregnancy
- Allergy or hypersensitivity to nitrates

For peer review only - http://bmjopen.bmj.com/site/about/guidelines.xhtml

- Consumption of alcohol in the last 24 hours
- Concomitant use of phosphodiesterase inhibitors
- Instrumental vaginal delivery in theatre
- Contra-indications to nitrates including severe anaemia, constrictive pericarditis, extreme bradycardia, incipient glaucoma, glusose-6-phosphate dehydrogenase–deficiency, cerebral haemorrhage and brain trauma, aortic and/or mitral stenosis, angina caused by hypertrophic obstructive cardiomyopathy, circulatory collapse, cardiogenic shock, and toxic pulmonary oedema

2.3 Recruitment and Study Procedures

2.3.1 Identifying participants

We initially followed the Royal College of Obstetrics and Gynaecology best practice recommendations for recruitment and obtaining consent for intrapartum research studies [19]. These recommendations were based on the work by Vernon et al, [20] who developed a consent pathway for women with retained placenta who were recruited to the RELEASE trial of umbilical vein oxytocin for RP [21]. We refined this pathway in light of our qualitative research [17, 18] so that the consent and recruitment pathways were tailored to the specific requirements of this trial. In brief, where possible, outline information about the trial (including researcher contact details) is made available to women antenatally, particularly those identified as being at high risk of retained placenta. More detailed information is available in antenatal clinics, on labour wards and the study website with information posters in the hospital reception areas and antenatal clinics. Following diagnosis of a retained placenta, a clinician or midwife who has received GOT-IT study training (which has sponsor approved GCP-training embedded within it), invites women to participate in the trial, provides an explanation of the study and gives potential participants an abbreviated and/or full version of the patient information sheet.

For peer review only - http://bmjopen.bmj.com/site/about/guidelines.xhtml

2.3.2 Informed Consent

The patient information leaflets explain that the trial is investigating whether sublingual GTN spray is useful for treating retained placenta. If a woman wishes to participate then written or verbal consent is obtained. If verbal consent is obtained, this is followed up by written consent as soon as possible in the postnatal period. Such an approach has been endorsed by the Royal College of Obstetricians and Gynaecologists in recent guidance about obtaining consent in perinatal research where consent is time critical in which it is stated that '[in acute circumstances]…provision of antenatal information to women with brief oral consent at the time of the complication is appropriate. Full written consent is then obtained at a later stage' [22]. Permission is sought to inform the woman's general practitioner that she is taking part in this trial. Due to the emergency nature of the condition (retained placenta), women are eligible to take part if they fulfil the inclusion criteria, understand what trial participation involves and give fully informed consent. A minimum time period is not feasible for trial consideration by potential participants. If a woman cannot give informed consent (e.g. due to incapacity), she is not eligible for participation.

2.3.3 Randomisation and allocation

Study drugs are prepared and kept on the delivery suite in pre-packed boxes. There is no requirement for refrigeration. After consent of each participant, the investigator or trained clinical practitioner randomises eligible and consenting women to one of the two study groups by taking the next box off the shelf. After priming the pump and demonstrating its use, the clinician gives the study drug to the women to self-administer. The study is double masked so neither the participant, investigator nor trained clinical practitioner know which treatment has been allocated. The boxes are numbered and labelled so that the identity of the drug can be determined later. The number on the box is logged in the study datbase. Breaking of the study masking is only performed where knowledge of the treatment is absolutely necessary for safe management of the patient.

For peer review only - http://bmjopen.bmj.com/site/about/guidelines.xhtml

A Central Emergency unblinding interactive voice response system is operated by the Centre for Healthcare Randomised Trials (ChaRT) in Aberdeen. Only senior clinicians can unblind trial participants. The name of the clinician requesting the unblinding, the reasons for it and notification of any unblinding is sent to the Chief Investigator via e-mail. Reasons for unblinding are recorded in the participant's sources documents and in the Investigator Site Master File. Unless there is a clinical requirement, masking will not be broken until after all study data entry is complete, the validity of the data is checked, all queries resolved and the patient populations agreed.

2.3.4 Follow-up procedures

Baseline observations (maternal blood pressure (mmHg) and heart rate (beats per minute)) are taken prior to study drug administration to confirm that the woman fulfils the haemodynamic inclusion criteria (heart rate ≤119 bpm, and systolic blood pressure >100mmHg) for trial entry.

Following confirmation of eligibility, women self-administer two puffs of either GTN (800mcg) or placebo spray as a once-only, sub-lingual intervention via a pump spray. Maternal temperature and a blood sample to measure haemoglobin are measured at baseline. Heart rate, systolic blood pressure and temperature (^{o}C) are recorded at five and 15 minutes after administration of the study drug.

Sublingual GTN is maximally effective in causing uterine and cervical relaxation by five minutes post administration. Thus, if spontaneous delivery of the placenta is going to occur, we expect this to happen around five minutes after drug administration. If spontaneous placental delivery has not occurred, a further attempt to deliver the placenta by controlled cord traction is made. If the placenta remains undelivered 15 minutes after administration of the treatment we think that it is highly unlikely that it will deliver spontaneously thereafter – we consider that any further delay risks haemorrhage. Thus the decision that manual removal of placenta is needed (primary clinical outcome) is made and the participant transferred to theatre for the definitive management of manual removal of placenta as soon as possible. Additionally, if there are clinically significant side-effects (e.g. haemorrhage,

For peer review only - http://bmjopen.bmj.com/site/about/guidelines.xhtml

symptomatic maternal hypotension and/or tachycardia) before 15 minutes have elapsed after GTN/placebo treatment, the participant is transferred to theatre for immediate manual removal of placenta.

On the first post-natal day a haemoglobin sample is collected. A questionnaire relating to the woman's perception of the study drug in relation to side-effects and patient satisfaction is also completed. A second questionnaire is sent by the Central Trial Office to participants at six weeks post-delivery. The questionnaire collects further information on patient-rated side-effects, patient rated satisfaction and health economic resource use.

Women remain in the trial unless they are unable to continue for a clinical reason or choose to withdraw consent. All other changes in status, with the exception of formal withdrawal of consent, mean the participant will be followed up for all study outcomes whenever possible.

2.4 Safety

The laws that govern reporting for Clinical Trials of Investigational Medicinal Products regulate safety reporting for the GOT-IT trial. Adverse events may occur during or after participation in the trial.

2.4.1 Definitions

An **adverse event** (AE) is any untoward medical occurrence in a clinical trial participant which does not necessarily have a causal relationship with an investigational medicinal product (IMP). Each AE is considered for severity, causality or expectedness and may be reclassified as a serious event.

For peer review only - http://bmjopen.bmj.com/site/about/guidelines.xhtml

An **adverse reaction** (AR) is any untoward and unintended response to the IMP which is related to any dose administered to that participant.

A **serious adverse event** (SAE), **serious adverse reaction** (SAR) is any AE or AR that at any dose:

- results in death of the clinical trial participant;
- is life threatening, defined as an event where the participant was at risk of death at the time of the event. It does not refer to an event which hypothetically might have caused death if it were more severe;
- requires in-patient hospitalisation or prolongation of existing hospitalisation;
- results in persistent or significant disability or incapacity;
- consists of a congenital anomaly or birth defect;
- results in any other significant medical event not meeting the criteria above.

A suspected unexpected serious adverse reaction (SUSAR) is any AR that is classified as serious and is suspected to be caused by the IMP, that it is not consistent with the information about the IMP in the summary of the medicinal product characteristics (SmPC).

2.4.2 Identifying AEs and SAEs

All AEs and SAEs are recorded from the time a participant signs the consent form to take part in the study until the six week postnatal outcome assessment point.

Participants are asked about the occurrence of AEs/SAEs prior to discharge from the hospital and in the six week postnatal questionnaire. Open-ended and non-leading verbal questioning of the

For peer review only - http://bmjopen.bmj.com/site/about/guidelines.xhtml

participant are used to enquire about AE/SAE occurrence at discharge. The six week postnatal questionnaire also asks participants if they have seen their general practitioner, been admitted to hospital, or been prescribed any medication. If there is any doubt as to whether a clinical observation is an AE, the event is recorded. AEs and SAEs are also identified via information from support departments e.g. laboratories.

2.4.3 Recording AEs and SAEs

When an AE/SAE occurs, it is the responsibility of the Principal Investigator to review all documentation (e.g. hospital notes, laboratory and diagnostic reports) related to the event. The Principal Investigator records all relevant information on the SAE form (if the AE meets the criteria of SAE). Information to be collected includes type of event, onset date, Investigator assessment of severity and causality, date of resolution as well as treatment required, investigations needed and outcome.

The clinician will assess all reported SAEs. The events detailed in the list below are 'expected' and will not be reported to the Co-Sponsors as an SAE but will be recorded in the case report form as a hospitalisation or outcome and presented to the Data Monitoring Committee, as part of the ongoing safety review.

For peer review only

In this trial, the following events are not considered SAEs:

i) Pregnancy is not considered an AE or SAE, as it is part of the inclusion criteria.

ii) Hospitalisations for treatment planned prior to randomisation and hospitalisation for elective treatment of a pre-existing condition will not be considered as an SAE. This includes pregnancy.

For peer review only - http://bmjopen.bmj.com/site/about/guidelines.xhtml

iii) Fall in haemoglobin of more than 15% between recruitment and the first postnatal day

iv) Manual removal of placenta in theatre.

v) Need for earlier than planned manual removal of placenta on the basis of the clinical condition.

vi) Fall in systolic or diastolic blood pressure of more than 15mmHg and/or increase in pulse of more than 20 beats/minute between baseline and five and 15 minutes post-administration of active/placebo treatment.

vii) Need for blood transfusion between time of delivery and discharge from hospital.

viii) Need for general anaesthesia.

ix) Maternal pyrexia (one or more temperature reading of more than 38^0C within 72 hours of delivery or discharge from hospital if discharge occurs sooner).

x) Sustained uterine relaxation after removal of placenta requiring uterotonics.

2.4.4 Assessment of AEs and SAEs

Seriousness, causality, severity and expectedness of each AE are assessed by the Principal Investigator. AEs are assessed as though the participant is taking active IMP. Cases that are considered serious, possibly, probably or definitely related to IMP and unexpected (i.e. SUSARs) are unblinded. The Chief Investigator may not downgrade an event that has been assessed by an Investigator as an SAE or SUSAR, but can upgrade an AE to an SAE, SAR or SUSAR if appropriate.

Assessment of Seriousness

The Investigator makes an assessment of seriousness as defined in 2.4.1.

Assessment of Causality

For peer review only - http://bmjopen.bmj.com/site/about/guidelines.xhtml

The Investigator makes an assessment of whether the AE/SAE is likely to be related to the IMP according to the definitions below.

- Unrelated: where an event is not considered to be related to the IMP.
- Possibly Related: The nature of the event, the underlying medical condition, concomitant medication or temporal relationship make it possible that the AE has a causal relationship to the study drug. The assessment of causality is made against the reference safety information found in the SmPC.

Where non Investigational Medicinal Products (NIMPs) e.g. rescue/escape drugs are given: if the AE is considered to be related to an interaction between the IMP and the NIMP, or where the AE might be linked to either the IMP or the NIMP but cannot be clearly attributed to either one of these, the event will be considered as an AR. Alternative causes such as natural history of the underlying disease, other risk factors and the temporal relationship of the event to the treatment are considered and investigated, however the blind will not be broken for the purpose of making this assessment.

Assessment of Expectedness

If an event is judged to be an AR, the evaluation of expectedness will be made based on knowledge of the reaction and the relevant product information documented in the SmPC. Events are classed as either expected: (the AR is consistent with the toxicity of the IMP listed in the SmPC) or Unexpected: (the AR is not consistent with the toxicity in the SmPC).

Assessment of Severity

The Investigator makes an assessment of severity for each AE/SAE and records this on the SAE form according to one of the following categories:

- Mild: an event that is easily tolerated by the participant, causing minimal discomfort and not interfering with every day activities.
- Moderate: an event that is sufficiently discomforting to interfere with normal everyday activities.

For peer review only - http://bmjopen.bmj.com/site/about/guidelines.xhtml

- Severe: an event that prevents normal everyday activities.

2.4.5 Reporting of SAEs/SAR/SUSARs

Once the Investigator becomes aware that an SAE has occurred in a study participant, the information is reported to the Academic and Clinical Central Office for Research and Development (ACCORD) Research Governance and Quality Assurance Office (Edinburgh University and NHS Lothian). immediately or within 24 hours. If the Investigator does not have all information regarding an SAE, they should not wait for this additional information before notifying ACCORD. The SAE report form can be updated when the additional information is received.

The SAE report provides an assessment of causality and expectedness at the time of the initial report to ACCORD. Where missing information has not been sent to ACCORD after an initial report, ACCORD contacts the Investigator and requests the missing information until this is supplied.

All reports sent to ACCORD and any follow up information are retained by the Investigator in the Investigator Site File. ACCORD also inform the trial office who check that the SAE is added to the CHaRT database.

2.4.6 Regulatory reporting requirements

The ACCORD Research Governance & QA Office is responsible for pharmacovigilance reporting on behalf of the co-sponsors (Edinburgh University and NHS Lothian). The ACCORD Research Governance and Quality Assurance Office has a legal responsibility to notify the regulatory competent authority and the Research Ethics Committee that approved the trial. Fatal or life threatening SUSARs are reported no later than seven calendar days and all other SUSARs are reported no later than 15 calendar days after ACCORD is first aware of the reaction. ACCORD informs Investigators at participating sites of all SUSARs and any other arising safety information. An Annual Safety Report/Development Safety Update Report is submitted, by ACCORD, to the regulatory authorities and Research Ethics Committees listing all SARs and SUSARs.

For peer review only - http://bmjopen.bmj.com/site/about/guidelines.xhtml

2.4.7 Follow up procedures

After initially recording an AE or recording and reporting an SAE, the Investigator follows each participant until resolution or death of the participant. Follow up information on an SAE is reported to the ACCORD office. AEs still present in participants at the six week postnatal outcome assessment point are monitored until resolution of the event or until no longer medically indicated.

2.5 Outcome Measures

The GOT-IT trial is assessing whether sublingual GTN spray is clinically and cost effective in treating retained placenta after vaginal delivery, reducing the need for manual removal of placenta following failure of current management.

2.5.1 Primary outcome measures

The primary outcome is measured over four interrelated domains of clinical, safety, patient-sided and economic.

The primary clinical outcome is need for manual removal of placenta, defined as the placenta remaining undelivered 15 minutes post study treatment and/or being required within 15 minutes of treatment due to safety concerns.

The primary safety outcome is measured blood loss between administration treatment and transfer to the postnatal ward or other clinical area.

The primary patient-sided outcome is satisfaction with treatment, a side effect profile assessed by a questionnaire administered at discharge from hospital and a postal questionnaire sent to the participant six weeks post hospital discharge.

For peer review only

For peer review only - http://bmjopen.bmj.com/site/about/guidelines.xhtml

The primary economic outcome is net incremental costs (or cost savings) to the National Health Service of using GTN versus standard practice. Costs include GTN (dose and time to administer drug), monitoring of the woman and delivering the placenta if effective), manual removal of placenta if required, and further health service resource use to six weeks post discharge.

2.5.2 Secondary Outcomes

The secondary endpoints are measured over two interrelated domains of clinical and economic outcomes. There are no secondary patient-sided or safety outcomes.

The secondary clinical endpoints include: i) Fall in haemoglobin of more than 15% between recruitment and the first postnatal day; ii) time from randomisation to delivery of placenta; iii) manual removal of placenta in theatre; iv) need for earlier than planned manual removal of placenta on the basis of the clinical condition, v) fall in systolic or diastolic blood pressure of more than 15mm Hg and/or increase in pulse of more than 20 beats/minute between baseline and five and 15 minutes post-administration of active/placebo treatment; vi) need for blood transfusion between time of delivery and discharge from hospital; vii) need for general anaesthesia; viii) maternal pyrexia (one or more temperature reading of more than 38°C within 72 hours of delivery or discharge from hospital if discharge occurs sooner); ix) sustained uterine relaxation after removal placenta requiring uterotonics; x) readmission to hospital for any reason within six weeks postpartum.

Estimated health service costs will be compared with multiple trial outcomes in a cost-consequence analysis of GTN versus standard practice. The costs and consequences of the alternative approaches to treatment will be summarised using a balance sheet, highlighting the costs and outcomes favouring each treatment option.

2.6 Data Collection and Processing

2.6.1 Measuring outcomes

For peer review only - http://bmjopen.bmj.com/site/about/guidelines.xhtml

The primary clinical outcome (need for manual removal of placenta, defined as the placenta remaining undelivered 15 minutes post treatment and/or being required within 15 minutes of treatment due to safety concerns) is collected 15 minutes post administration of the study drug. The primary safety outcome (measured blood loss between administration treatment and transfer to the postnatal ward/other clinical area) is recorded immediately prior to transfer from theatre to the postnatal ward or other clinical area (e.g. labour ward high dependency). The primary patient satisfaction outcome is collected at six weeks by a self-completed satisfaction questionnaire and the primary economic outcome will be computed from costs incurred at initial management of retained placenta and management of further complications and self-reported health service usage and side-effects recorded from the postnatal patient questionnaires.

Outcome data is collected on electronic case report forms and paper-based questionnaires. Health service use in the six weeks following discharge is collected via a small number of resource use questions included in the postnatal questionnaires. Resource use will be evaluated using routine sources of nationally relevant unit costs. Women are permitted to receive all standard care medical treatments for postpartum complications including any given for medical management of retained placenta. Concomitant medications are recorded from 24 hours prior to signing of the consent forms until 24 hours after administration of the study drug. Analgesic or antibiotic medications are recorded from 24 hours prior to signing of informed consent until six weeks after administration of study drug.

2.7 Sample Size, proposed recruitment rate and milestones

2.7.1 Sample size

The trial uses a group sequential design as opposed to a fixed sample design to allow for the uncertainty of two critical factors. First, there is considerable uncertainty about how many women who are eligible for the trial will actually go on and require surgery due to a) lack of knowledge of the frequency of spontaneous delivery of the placenta beyond the time frame that GTN is known to have a pharmacological effect, and b) the effect of variations in local clinical practice in relation to

For peer review only - http://bmjopen.bmj.com/site/about/guidelines.xhtml

organising manual removal of placentas. Secondly, the magnitude of the benefit from GTN spray is unknown. To properly reflect these uncertainties, we are using a group sequential design. This design maximises our chances of efficiently detecting and estimating the true benefit of treatment in the quickest time with the right number of participants, and gives us the opportunity to abandon the trial if it turns out that no worthwhile treatment effect exists (via futility analyses).

In discussion with clinicians and women, a minimally clinically important difference of an absolute reduction of 10% in the need for manual removal of placenta would need to be observed in order to accommodate this intervention (sublingual GTN) in clinical practice. Given that the maximum variability in a binary outcome (yes or no) occurs at a 50% rate in the placebo spray arm, a fixed sample approach would need 1038 women (519 in each group) to demonstrate a 10% change from 50% on placebo to 40% on GTN spray. As the outcome (surgery occurred – yes or no) takes place 15 minutes after the intervention has been administered there will be minimal loss to follow-up and therefore, no requirement for a trial much larger than this. Using a group sequential design incorporating a Lan-DeMets alpha spending function with O'Brien-Fleming boundaries, our trial has a maximum sample size of 1086 (with a small inflation to 1100 to allow for withdrawal of consent of other rare loss to follow-up). This allows for five planned interim evaluations by the Data Monitoring Committee of accruing data at 218, 436, 652 and 870 participants with a final look (if the trial proceeds to full size) at 1086 women.

2.8 Analyses Plan

2.8.1 Statistical Analysis

The statistical analyses will be according to the Intention-To-Treat Principle and will be governed by a Statistical Analysis Plan. The interim analyses will be specified within the Data Monitoring Committee Charter and the result of the interim analyses will be in strict confidence and unknown to the trial research group apart from the study statistician. The primary outcomes: clinical (manual

For peer review only - http://bmjopen.bmj.com/site/about/guidelines.xhtml

removal of placenta), safety (blood loss), patient satisfaction and economic (NHS costs) will be analysed using linear models appropriate to the distribution of each outcome. These models will adjust for relevant baseline factors, and sensitivity analyses will assess the robustness of the findings to any missing data (which is expected to be very low as the primary outcome is measured within minutes of receiving the intervention). Subgroup analyses of the primary outcome will be explored.

2.8.2 Economic Evaluation

While the cost of GTN is low, there will still be costs associated with its administration and the monitoring and management of women thereafter. Should the intervention prove effective, it will be important in the context of scarce maternity resources to explicitly quantify the net costs (or cost savings) associated with its use. We have therefore made provision to carry out a simple cost analysis using clinical and resource use data which is being collected for individual participants recruited to the trial. This analysis will explicitly quantify the difference in mean costs between the active intervention and placebo arm. Research costs associated with placebo delivery will be factored out of the analysis to estimate the incremental cost (or cost savings) of the active intervention versus standard practice.

Resource use associated with the alternative management strategies will be estimated from the time of randomisation through to six weeks post-partum. This will include: 1) staff time for administering the drug to patients; 2) resource use associated with any complications arising following administration of the study drug (e.g. blood pressure and/or heart rate monitoring); 3) subsequent costs associated with delivery of the placenta (either spontaneously or operatively); and 4) subsequent health service contact relating to retained products of conception up to six weeks post discharge.

For peer review only

For peer review only - http://bmjopen.bmj.com/site/about/guidelines.xhtml

The mean costs will be summarised by treatment allocation group, and the incremental cost (or cost saving) associated with the use of GTN will be estimated using an appropriately specified general linear model. The cost data will be presented alongside the primary and secondary outcome data in a cost-consequence balance sheet, indicating which strategy each outcome favours.

2.9 Organisation: Trial Management, Oversight and Governance Arrangements

2.9.1 Trial management

The trial is being jointly run by CHaRT based at the University of Aberdeen, the Chief Investigator and the ACCORD office at the University of Edinburgh. The trial management and governance are as recommended by The Research Governance Framework and The Medicines for Human Use (Clinical Trials) Regulations 2004. Briefly, we have set up a Trial Steering Committee and a Data Monitoring Committee led by individuals independent of the project and the institutions involved. The membership of the Trial Steering Committee includes independent members including a lay representative. A separate and independent Data Monitoring Committee has also been convened. The Data Monitoring Committee met during trial set-up to agree terms of reference and is meeting a further five times during recruitment to monitor accumulating data and oversee safety issues. The Data Monitoring Committee will meet one final time after unblinding to assess overall data validity and analyses. The Trial Steering Committee is meeting approximately seven times with the meeting spacing being matched to that of the Data Monitoring Committee. Finally, we have regular Project Management Group meetings and Research Midwives Meetings to ensure consistency of trial processes across sites and share best practice for recruitment, consent and data collection.

2.9.2 Research governance and compliance

For peer review only - http://bmjopen.bmj.com/site/about/guidelines.xhtml

2.9.2.1 Research Governance

ACCORD is providing oversight that the appropriate research and competent authority approvals are in place, pharmacovigilance services and is ensuring the trial is conducted in accordance with the principles of the International Conference on Harmonisation Tripartite Guideline for Good Clinical Practice.

2.9.2.2 Data protection

The trial complies with the Data Protection Act 1998 and regular monitoring and checks are in place to ensure compliance. Data is being stored in accordance with the Act and will be archived to a secure data storage facility. The consent form states that other researchers may wish to access (anonymised) data in the future. The Senior Information Technology Manager (in collaboration with the trial statistician and trial manager) will manage access rights to the data set. Prospective new users must demonstrate compliance with data protection, legal and ethical guidelines before any data are released. We anticipate that anonymised trial data will be shared with other researchers to enable international prospective meta-analyses.

2.9.2.3 Sponsorship

The University of Edinburgh and NHS Lothian are the co-sponsors for the trial. Co-sponsorship responsibilities are detailed in an agreement. Responsibilities sub-contracted to CHaRT are also detailed in an agreement which detail the responsibilities surrounding centralised trial administration, database support and economic and statistical analyses.

2.9.2.3 Quality Assurance

For peer review only - http://bmjopen.bmj.com/site/about/guidelines.xhtml

For peer review only

An independent risk assessment has been performed to determine the level of monitoring required and if an audit should be performed before/during/after the study and if so, at what locations and at what frequency.

2.9.2.4 Data Handling, Record Keeping and Archiving

The local principal investigator or research midwives enter locally-collected data from the study centres into the web-based data collection application. The 6-week follow-up questionnaires are sent from and returned to the Study Office in Edinburgh and are inputted by the staff in the Study Office in Edinburgh. CHaRT is working with the Study Office in Edinburgh and with local Research Midwives to ensure that the data is as complete and accurate as possible.

3.0 Ethics, Regulatory Approvals and Dissemination

3.1 Ethics and Regulatory Approvals

The Trial is registered on the International Standard Randomised Controlled Trial Number (ISRCTN) as ISCRTN88609453. Ethical approval has been obtained from the North East- Newcastle & North Tyneside 2 Research Ethics Committee [13/NE/0339]. Approval has also been obtained from the Medicines and Healthcare Products Regulatory Authority (MHRA) [2013-003819-42] and the Health Research Authority as well as approvals from the local Trust Research and Development Offices. The study has been adopted to the National Institute for Health Research Clinical Research Network (NIHR CRN) Portfolio [http://www.nihr.ac.uk/research-and-impact/nihr-clinical-research-network-portfolio/]. The necessary trial insurance is provided by the University of Edinburgh.

3.2 Dissemination

For peer review only - http://bmjopen.bmj.com/site/about/guidelines.xhtml

The dissemination plans include the Health Technology Assessment monograph, presentation at international scientific meetings, and publication of the results of the main trial and each of the secondary endpoints in high impact peer reviewed journals. If all grant-holders and researcher staff fulfil authorship rules, group authorship will be used under the collective title of ‘the GOT-IT Trial Group’. The Chief Investigator, Trial Manager, and possibly other members of the trial group will take responsibility for drafting the paper and this will be recognised by line ‘the Chief Investigator (as primary author), followed by the other authors and the GOT-IT Trial Group'. Authorship of the qualitative sub-study publications [17, 18] has been attributed to Chief Investigator and the named individual(s). To maintain interest in the study, we are also publishing GOT-IT newsletters at intervals to trial staff and collaborators.

For peer review only

For peer review only - http://bmjopen.bmj.com/site/about/guidelines.xhtml

4.0 References:

1. NICE, *NICE clinical guideline 55: Intrapartum care: care of healthy women and their babies during childbirth.* 2012.
2. Stones, R.W., C.M. Paterson, and N.J. Saunders, *Risk factors for major obstetric haemorrhage.* Eur J Obstet Gynecol Reprod Biol, 1993. **48**(1): p. 15-8.
3. Combs, C.A. and R.K. Laros, Jr., *Prolonged third stage of labor: morbidity and risk factors.* Obstet Gynecol, 1991. **77**(6): p. 863-7.
4. Cheung, W.M., et al., *The retained placenta: historical and geographical rate variations.* J Obstet Gynaecol, 2011. **31**(1): p. 37-42.
5. Ely, J.W., et al., *The association between manual removal of the placenta and postpartum endometritis following vaginal delivery.* Obstet Gynecol, 1995. **86**(6): p. 1002-6.
6. Combs, C.A., E.L. Murphy, and R.K. Laros, Jr., *Factors associated with postpartum hemorrhage with vaginal birth.* Obstet Gynecol, 1991. **77**(1): p. 69-76.
7. Abdel-Aleem, H., M.A. Abdel-Aleem, and O.M. Shaaban, *Tocolysis for management of retained placenta.* Cochrane Database Syst Rev, 2011(1): p. CD007708.
8. Weeks, A.D., *The retained placenta.* Best Pract Res Clin Obstet Gynaecol, 2008. **22**(6): p. 1103-17.
9. Farley, A.E., C.H. Graham, and G.N. Smith, *Contractile properties of human placental anchoring villi.* Am J Physiol Regul Integr Comp Physiol, 2004. **287**(3): p. R680-5.
10. Barnes, F., *Hour-Glass Contraction of the Uterus Treated with Nitrite of Amyl.* Br Med J, 1882. **1**(1107): p. 377.
11. Bullarbo, M., J. Tjugum, and E. Ekerhovd, *Sublingual nitroglycerin for management of retained placenta.* Int J Gynaecol Obstet, 2005. **91**(3): p. 228-32.
12. Redick, L.F. and E. Livingston, *A new preparation of nitroglycerin for uterine relaxation.* Int J Obstet Anesth, 1995. **4**(1): p. 14-6.
13. Laslett, L.J. and L. Baker, *Sublingual nitroglycerin administered by spray versus tablet: comparative timing of hemodynamic effects.* Cardiology, 1990. **77**(4): p. 303-10.
14. Marmor, A., *Comparative evaluation of a new formulation of isosorbide dinitrate oral spray and sublingual nitroglycerin tablets.* Am J Cardiol, 1990. **65**(21): p. 43J-45J.
15. Curry, S.H., et al., *Plasma concentrations and hemodynamic effects of intravenous, sublingual, and aerosolized nitroglycerin in patients undergoing cardiac catheterization.* Biopharm Drug Dispos, 1993. **14**(2): p. 107-18.
16. Wight, L.J., et al., *A large scale comparative study in general practice with nitroglycerin spray and tablet formulations in elderly patients with angina pectoris.* Eur J Clin Pharmacol, 1992. **42**(3): p. 341-2.
17. Hallowell, N., et al., *The role of therapeutic optimism in recruitment to a clinical trial in a peripartum setting: balancing hope and uncertainty.* Trials, 2016. **17**(1): p. 267.
18. Lawton, J., et al., *Recruiting and consenting into a peripartum trial in an emergency setting: a qualitative study of the experiences and views of women and healthcare professionals.* Trials, 2016. **17**(1): p. 195.
19. RCOG, *Obtaining valid consent to participate in research while in labour*, in *Clinical Governance Advice No.6a*2010, RCOG: London.
20. Vernon, G., Z. Alfirevic, and A. Weeks, *Issues of informed consent for intrapartum trials: a suggested consent pathway from the experience of the Release trial [ISRCTN13204258].* Trials, 2006. **7**: p. 13.
21. Weeks, A.D., et al., *Umbilical vein oxytocin for the treatment of retained placenta (Release Study): a double-blind, randomised controlled trial.* Lancet, 2010. **375**(9709): p. 141-7.
22. RCOG, *Obtaining Valid Consent to Participate in Perinatal Research Where Consent is Time Critical*, in *Clinical Governance Advice no. 6a*, ROCG, Editor 2016, RCOG: London.

For peer review only - http://bmjopen.bmj.com/site/about/guidelines.xhtml

Authors' contributions:

FCD conceived and designed the study. FCD, JN, JL, GS, GM, AM, JB, MP, CC, SBS, NH and JEN drafted the original grant proposal and trial protocol. JN and JH provide methodological and statistical expertise. JL and NH provide expertise in the qualitative studies. FCD, JEN and SBS provide expertise in the clinical outcomes. KFC and SM have drafted protocol revisions and have both been trial managers for the trial. MF and AM provide database and trial support from the trials unit. JB and CC provide lay input. FCD and KFC drafted the manuscript. KFC has responsibilities for day-to-day running of the trial including participant recruitment, data collection and liaising with other sites. All authors critically reviewed and approved the final version of the manuscript.

Funding statement:

The GOT-IT Trial (trial registration number: ISCRTN 88609453) is funded by the National Institute for Health Research Health Technology Assessment (HTA) programme (Project number 12/29/01). The views and opinions expressed therein are those of the authors and do not necessarily reflect those of the HTA, the National Institute for Health Research, the NHS or the Department of Health. This work was undertaken in the MRC Centre for Reproductive Health which is funded by MRC Centre grant (MRC G1002033).

Competing interests statement:

The authors have no competing interests to declare.

For peer review only

For peer review only - http://bmjopen.bmj.com/site/about/guidelines.xhtml

SPIRIT

STANDARD PROTOCOL ITEMS: RECOMMENDATIONS FOR INTERVENTIONAL TRIALS

SPIRIT 2013 Checklist: Recommended items to address in a clinical trial protocol and related documents*

Section/item	Item No	Description	Addressed on page number
**Administrative information**			
Title	1	Descriptive title identifying the study design, population, interventions, and, if applicable, trial acronym	Page 1
Trial registration	2a	Trial identifier and registry name. If not yet registered, name of intended registry	Page 3
	2b	All items from the World Health Organization Trial Registration Data Set	/
Protocol version	3	Date and version identifier	Page
Funding	4	Sources and types of financial, material, and other support	Page 3
Roles and responsibilities	5a	Names, affiliations, and roles of protocol contributors	Page 1
	5b	Name and contact information for the trial sponsor	Page 17
	5c	Role of study sponsor and funders, if any, in study design; collection, management, analysis, and interpretation of data; writing of the report; and the decision to submit the report for publication, including whether they will have ultimate authority over any of these activities	Page 17
	5d	Composition, roles, and responsibilities of the coordinating centre, steering committee, endpoint adjudication committee, data management team, and other individuals or groups overseeing the trial, if applicable (see Item 21a for data monitoring committee)	Page 23

For peer review only

For peer review only - http://bmjopen.bmj.com/site/about/guidelines.xhtml

Introduction

Background and rationale	6a	Description of research question and justification for undertaking the trial, including summary of relevant studies (published and unpublished) examining benefits and harms for each intervention	Pages 4 & 5
	6b	Explanation for choice of comparators	Pages 4 & 5
Objectives	7	Specific objectives or hypotheses	Pages 5 & 6
Trial design	8	Description of trial design including type of trial (eg, parallel group, crossover, factorial, single group), allocation ratio, and framework (eg, superiority, equivalence, noninferiority, exploratory)	Pages 5 & 6

Methods: Participants, interventions, and outcomes

Study setting	9	Description of study settings (eg, community clinic, academic hospital) and list of countries where data will be collected. Reference to where list of study sites can be obtained	Pages 7 & 8
Eligibility criteria	10	Inclusion and exclusion criteria for participants. If applicable, eligibility criteria for study centres and individuals who will perform the interventions (eg, surgeons, psychotherapists)	Pages 8 & 9
Interventions	11a	Interventions for each group with sufficient detail to allow replication, including how and when they will be administered	Pages 10 & 11
	11b	Criteria for discontinuing or modifying allocated interventions for a given trial participant (eg, drug dose change in response to harms, participant request, or improving/worsening disease)	Page 11
	11c	Strategies to improve adherence to intervention protocols, and any procedures for monitoring adherence (eg, drug tablet return, laboratory tests)	Pages 11
	11d	Relevant concomitant care and interventions that are permitted or prohibited during the trial	Page 11
Outcomes	12	Primary, secondary, and other outcomes, including the specific measurement variable (eg, systolic blood pressure), analysis metric (eg, change from baseline, final value, time to event), method of aggregation (eg, median, proportion), and time point for each outcome. Explanation of the clinical relevance of chosen efficacy and harm outcomes is strongly recommended	Page 11, 18 - 20
Participant timeline	13	Time schedule of enrolment, interventions (including any run-ins and washouts), assessments, and visits for participants. A schematic diagram is highly recommended (see Figure)	Page 11, 18 - 20

For peer review only - http://bmjopen.bmj.com/site/about/guidelines.xhtml

Sample size	14	Estimated number of participants needed to achieve study objectives and how it was determined, including clinical and statistical assumptions supporting any sample size calculations	Pages 20 & 21
Recruitment	15	Strategies for achieving adequate participant enrolment to reach target sample size	Pages 20 & 21
**Methods: Assignment of interventions (for controlled trials)**			
Allocation:			
Sequence generation	16a	Method of generating the allocation sequence (eg, computer-generated random numbers), and list of any factors for stratification. To reduce predictability of a random sequence, details of any planned restriction (eg, blocking) should be provided in a separate document that is unavailable to those who enrol participants or assign interventions	Pages 10 & 11
Allocation concealment mechanism	16b	Mechanism of implementing the allocation sequence (eg, central telephone; sequentially numbered, opaque, sealed envelopes), describing any steps to conceal the sequence until interventions are assigned	Pages 10 & 11
Implementation	16c	Who will generate the allocation sequence, who will enrol participants, and who will assign participants to interventions	Pages 10 & 11
Blinding (masking)	17a	Who will be blinded after assignment to interventions (eg, trial participants, care providers, outcome assessors, data analysts), and how	Pages 10 & 11
	17b	If blinded, circumstances under which unblinding is permissible, and procedure for revealing a participant's allocated intervention during the trial	Pages 10 & 11
**Methods: Data collection, management, and analysis**			
Data collection methods	18a	Plans for assessment and collection of outcome, baseline, and other trial data, including any related processes to promote data quality (eg, duplicate measurements, training of assessors) and a description of study instruments (eg, questionnaires, laboratory tests) along with their reliability and validity, if known. Reference to where data collection forms can be found, if not in the protocol	Pages 18 - 20
	18b	Plans to promote participant retention and complete follow-up, including list of any outcome data to be collected for participants who discontinue or deviate from intervention protocols	Pages 18 - 20

For peer review only - http://bmjopen.bmj.com/site/about/guidelines.xhtml

Data management	19	Plans for data entry, coding, security, and storage, including any related processes to promote data quality (eg, double data entry; range checks for data values). Reference to where details of data management procedures can be found, if not in the protocol	Pages 18 - 20
Statistical methods	20a	Statistical methods for analysing primary and secondary outcomes. Reference to where other details of the statistical analysis plan can be found, if not in the protocol	Pages 20 - 22
	20b	Methods for any additional analyses (eg, subgroup and adjusted analyses)	Pages 20 - 22
	20c	Definition of analysis population relating to protocol non-adherence (eg, as randomised analysis), and any statistical methods to handle missing data (eg, multiple imputation)	Pages 20 - 23
**Methods: Monitoring**			
Data monitoring	21a	Composition of data monitoring committee (DMC); summary of its role and reporting structure; statement of whether it is independent from the sponsor and competing interests; and reference to where further details about its charter can be found, if not in the protocol. Alternatively, an explanation of why a DMC is not needed	Pages 24 - 25
	21b	Description of any interim analyses and stopping guidelines, including who will have access to these interim results and make the final decision to terminate the trial	Pages 20 - 25
Harms	22	Plans for collecting, assessing, reporting, and managing solicited and spontaneously reported adverse events and other unintended effects of trial interventions or trial conduct	Pages 20 - 25
Auditing	23	Frequency and procedures for auditing trial conduct, if any, and whether the process will be independent from investigators and the sponsor	Pages 20 - 25
**Ethics and dissemination**			
Research ethics approval	24	Plans for seeking research ethics committee/institutional review board (REC/IRB) approval	Page 25 - 26
Protocol amendments	25	Plans for communicating important protocol modifications (eg, changes to eligibility criteria, outcomes, analyses) to relevant parties (eg, investigators, REC/IRBs, trial participants, trial registries, journals, regulators)	Page 25 - 26

For peer review only - http://bmjopen.bmj.com/site/about/guidelines.xhtml

Consent or assent	26a	Who will obtain informed consent or assent from potential trial participants or authorised surrogates, and how (see Item 32)	Page 10
	26b	Additional consent provisions for collection and use of participant data and biological specimens in ancillary studies, if applicable	Not applicable
Confidentiality	27	How personal information about potential and enrolled participants will be collected, shared, and maintained in order to protect confidentiality before, during, and after the trial	Page 24
Declaration of interests	28	Financial and other competing interests for principal investigators for the overall trial and each study site	Page 3
Access to data	29	Statement of who will have access to the final trial dataset, and disclosure of contractual agreements that limit such access for investigators	Page 24 & 25
Ancillary and post-trial care	30	Provisions, if any, for ancillary and post-trial care, and for compensation to those who suffer harm from trial participation	Page 25
Dissemination policy	31a	Plans for investigators and sponsor to communicate trial results to participants, healthcare professionals, the public, and other relevant groups (eg, via publication, reporting in results databases, or other data sharing arrangements), including any publication restrictions	Page 25 and 26
	31b	Authorship eligibility guidelines and any intended use of professional writers	Page 25 and 26
	31c	Plans, if any, for granting public access to the full protocol, participant-level dataset, and statistical code	Not applicable
**Appendices**			
Informed consent materials	32	Model consent form and other related documentation given to participants and authorised surrogates	If requested
Biological specimens	33	Plans for collection, laboratory evaluation, and storage of biological specimens for genetic or molecular analysis in the current trial and for future use in ancillary studies, if applicable	Not applicable

*It is strongly recommended that this checklist be read in conjunction with the SPIRIT 2013 Explanation & Elaboration for important clarification on the items. Amendments to the protocol should be tracked and dated. The SPIRIT checklist is copyrighted by the SPIRIT Group under the Creative Commons "Attribution-NonCommercial-NoDerivs 3.0 Unported" license.

For review only

BMJ Open

A pragmatic group sequential placebo controlled randomised trial to determine the effectiveness of Glyceryl trinitrate for retained placenta (GOT-IT): a study protocol

Journal:	*BMJ Open*
Manuscript ID	bmjopen-2017-017134.R1
Article Type:	Protocol
Date Submitted by the Author:	29-May-2017

Complete List of Authors:	Denison, Fiona; The University of Edinburgh , MRC Centre for Reproductive Health<br>Norrie, John; Edinburgh Clinical Trials Unit, University of Edinburgh No. 9, Bioquarter<br>Lawton, Julia; University of Edinburgh, Centre for Population Health Sciences<br>Norman, Jane; University of Edinburgh, MRC Centre for Reproductive Health<br>Scotland, Graham; University of Aberdeen, Health Economics Research Unit<br>McPherson, Gladys; Health Services Research Unit Centre for Healthcare Randomised Trials Health Services Research Unit<br>McDonald, Alison; University of Aberdeen, The Centre for Healthcare Randomised Trials (CHaRT), Health Services Research Unit, Foresterhill<br>Forrest, Mark; The Centre for Healthcare Randomised Trials (CHaRT), Health Services Research Unit, University of Aberdeen, Foresterhill<br>Hudson, Jemma; The Centre for Healthcare Randomised Trials (CHaRT), Health Services Research Unit, University of Aberdeen, Foresterhill<br>Brewin, Jane; Tommy's,<br>Peace, Mathilde<br>Clarkson, Cynthia<br>Brook-Smith, Sheonagh; NHS Lothian, Simpson's Centre for Reproductive Health, Royal Infirmary of Edinburgh<br>Morrow, Susan; The University of Edinburgh, Asthma UK Centre for Applied Research, Centre for Medical Informatics, Usher Institute of Population Health Sciences and Informatics<br>Hallowell, Nina; Ethox Centre, Nuffield Department of Population Health, University of Oxford, Old Road<br>Hodges, Laura; University of Edinburgh, MRC Centre for Reproductive Health Edinburgh<br>Carruthers, Kathryn; University of Edinburgh, Centre for Cardiovascular Science

**Primary Subject Heading**:	Obstetrics and gynaecology
Secondary Subject Heading:	Obstetrics and gynaecology, Health economics, Evidence based practice
Keywords:	retained placenta, randomised controlled trial, Glyceryl trinitrate

SCHOLARONE™
Manuscripts

For peer review only

A pragmatic group sequential placebo controlled randomised trial to determine the effectiveness of Glyceryl trinitrate for retained placenta (GOT-IT): a study protocol

Denison FC[1]*, Norrie J[2], Lawton J[3], Norman JE[1], Scotland G[4], McPherson G[2], McDonald A[2], Forrest M[2], Hudson J[2], Brewin J[5], Peace M[1], Clarkson C[1], Brook-Smith S[6], Morrow S[7], Hallowell N[3,8], Hodges L[1], Carruthers KF[1]

* Corresponding author:

Fiona Denison: Fiona.Denison@ed.ac.uk

[1]Tommy's Centre for Maternal and Fetal Health Research, Medical Research Council Centre for Reproductive Health, Queen's Medical Research Institute, University of Edinburgh, 47 Little France Crescent, Edinburgh, UK

[2]The Centre for Healthcare Randomised Trials (CHaRT), Health Services Research Unit, University of Aberdeen, Foresterhill, Aberdeen, UK

[3]Centre for Population Health Sciences, University of Edinburgh, Edinburgh, UK

[4]Health Economics Research Unit, Institute of Applied Health Sciences, University of Aberdeen, Foresterhill, Aberdeen, UK

[5]Tommy's, Nicholas House, □3 Laurence Poutney Hill, London, UK

[6]Simpson's Centre for Reproductive Health, Royal Infirmary of Edinburgh, Edinburgh, UK

[7]Usher Institute of Population Health Sciences and Informatics, University of Edinburgh

[8]Ethox Centre, Nuffield Department of Population Health, University of Oxford, UK

For peer review only

Abstract:

Introduction

A retained placenta is diagnosed when the placenta is not delivered following delivery of the baby. It is a major cause of postpartum haemorrhage and treated by the operative procedure of manual removal of placenta (MROP).

Methods and analysis

The aim of this pragmatic randomised placebo controlled double blind UK-wide trial, with an internal pilot and nested qualitative research to adjust strategies to refine delivery of the main trial, is to determine whether sublingual GTN is (or is not) clinically and cost effective for (medical) management of retained placenta. The primary clinical outcome is need for MROP, defined as the placenta remaining undelivered 15 minutes post study treatment and/or being required within 15 minutes of treatment due to safety concerns. The primary safety outcome is measured blood loss between administration of treatment and transfer to the postnatal ward or other clinical area. The primary patient-sided outcome is satisfaction with treatment and a side effect profile. The primary economic outcome is net incremental costs (or cost savings) to the National Health Service of using GTN versus standard practice. Secondary outcomes are being measured over a range of clinical and economic domains. The primary outcomes will be analysed using linear models appropriate to the distribution of each outcome. Health service costs will be compared with multiple trial outcomes in a cost-consequence analysis of GTN versus standard practice.

Ethics and dissemination

Ethical approval has been obtained from the North-East Newcastle & North Tyneside 2 Research Ethics Committee [13/NE/0339]. Dissemination plans for the trial include the Health Technology Assessment Monograph, presentation at international scientific meetings, and publication in high impact peer reviewed journals.

Trial Registration

For peer review only - http://bmjopen.bmj.com/site/about/guidelines.xhtml

The trial is funded by the HTA (12/29/01) and has been registered on the International Standard Randomised Controlled Trial Number (ISRCTN) as ISCRTN88609453.

For peer review only

For peer review only - http://bmjopen.bmj.com/site/about/guidelines.xhtml

Strengths and limitations of this study

- Pragmatic randomised placebo controlled double blind UK-wide trial to determine whether sublingual GTN is (or is not) clinically and cost effective for (medical) management of retained placenta.
- Inclusion of clinical, patient-sided, safety and economic outcomes with minimal exclusion criteria.
- Pragmatic approach to obtaining consent and GCP-training to facilitate recruitment in the emergency setting
- Fully adaptive group sequential design
- Inability to differentiate whether a placenta is adherent or trapped prior to administration of study drug

For peer review only

For peer review only - http://bmjopen.bmj.com/site/about/guidelines.xhtml

1.0 INTRODUCTION

1.1 Background

A retained placenta is diagnosed when the placenta is not delivered within 30 or 60 minutes after delivery of the baby following active or physiological management of the third stage of labour, respectively [1] and is a major cause of postpartum haemorrhage [2, 3]. The incidence of retained placenta is rising in the UK due to changes in maternal demographics, increased intrapartum interventions and operations involving exploration of the uterine cavity including surgical termination of pregnancy and dilation and curettage [4]. Retained placenta currently affects approximately 2% of vaginal deliveries [4] which equates to nearly 11,000 women per annum in the UK. Following failure of active or physiological management, the National Institute of Health and Care Excellence recommends that retained placenta should be treated by the operative procedure of manual removal of the placenta [1]. This procedure requires skilled personnel, an operative environment and, compared to spontaneous placental delivery, has its own attendant risks, including bleeding [3] and infection [5]. There is often a delay in undertaking the operation, which leads to further haemorrhage [6], and the procedure itself is costly in terms of staff time. Furthermore, the infrastructure required for this operative intervention is not available in all delivery settings and the invasive nature of this procedure has the potential to delay or interrupt mother-baby bonding in the immediate post-partum period. To reduce the number of women requiring manual removal of placenta, there is therefore a need for new (and effective) medical treatments for retained placenta. The reduction in operative interventions would potentially have cost benefits for the National Health Service (NHS) and also for women in terms of increased satisfaction, less separation between mother and baby immediately after birth, and reduced morbidity.

1.2 Rationale for study

Observational studies along with results from small randomised controlled trials have suggested that nitric oxide donors including glyceryl trinitrate (GTN) given intravenously, might be effective for the

For peer review only

For peer review only - http://bmjopen.bmj.com/site/about/guidelines.xhtml

management of retained placenta with success rates ranging between 94% to 100% [7-10]. Although intravenous GTN appears to be efficacious for retained placenta in these small studies, this route of administration is not practical in all settings and intravenous administration causes unacceptable side-effects including symptomatic hypotension at higher doses [3]. More recently, sublingual administration has been trialed, with studies reporting experience with sublingual GTN tablet and spray. Using a sublingual GTN tablet, two small studies suggest a beneficial effect of sublingual GTN tablet; however a third larger study does not (RCT ISRCTN34755982; 37.3% vs 20.4%; GTN vs placebo; $p > 0.05$; n=105 women) [11]. The alternative preparation for sublingual administration is GTN spray [12]. Compared to the tablet preparation, sublingual GTN spray has several advantages including stability at room temperature, significant reduction in latency of onset with onset beginning at 30-45 seconds, peaking at 90-120 seconds and lasting up to five minutes [13-15], and fewer objective and subjective side-effects [16]. GTN spray is accepted obstetric management in other scenarios where rapid tocolysis is required such as uterine relaxation for release of a trapped head in breech delivery or at caesarean section [12]. Furthermore, anecdotal reports suggest that GTN spray may also have a use in management of retained placenta [12].

To summarise, although a growing body of evidence supports GTN use for the treatment of retained placenta, much of this evidence is based on anecdotal case-reports or clinical studies which are non-randomised, do not included a placebo arm and are underpowered. There is therefore a need to undertake a well designed randomised placebo controlled double blind pragmatic group sequential UK-wide (GOT-IT trial) to definitively determine whether sublingual GTN is (or is not) clinically and cost effective for management of retained placenta.

1.3 Study objectives

The aim of this placebo controlled, double blind, pragmatic group sequential UK-wide trial is to determine the effectiveness and cost-effectiveness of GTN for treating retained placenta and avoiding manual removal of placenta.

For peer review only - http://bmjopen.bmj.com/site/about/guidelines.xhtml

The hypothesis being tested is that sublingual GTN spray is clinically and cost effective in treating retained placenta after vaginal delivery, reducing the need for manual removal of placenta following failure of current management.

The primary objectives are to: 1) determine the clinical effectiveness of sublingual GTN in treating retained placenta and avoiding manual removal of placenta in women with vaginal delivery following failure of current management (clinical domain), 2) determine the side-effect profile for GTN given to treat retained placenta (safety domain), 3) assess patient satisfaction with GTN given for retained placenta (patient-sided domain) and 4) assess the net costs (or cost savings) to the National Health Service of using GTN for the treatment of retained placenta compared to standard practice (economic domain)

The secondary objectives are to assess NHS costs in relation to the primary outcome and range of secondary outcomes which are expected to differ between the two arms of the trial, using a cost-consequence balance sheet approach.

The trial contained an internal pilot, which commenced in October 2014 and was completed in April 2015. The aim of this internal pilot, which recruited from eight sites across the UK, was to provide reassurance on all the trial processes, including recruitment, consent, randomisation, delivery of treatment, and follow-up assessments to ensure that all were running smoothly. Recruitment to the substantive trial is ongoing and is due to finish in September 2017.

A nested qualitative study was undertaken during the internal pilot to adjust strategies to (a) maximise recruitment into the main trial; (b) refine the recruitment and consent pathway and (c) ensure staff are given appropriate training and support to help promote the successful delivery of the main trial. In-depth qualitative interviews were undertaken with staff (n=27) involved in trial recruitment, consenting and/or trial delivery and with trial recruits (n=22) within, when possible, four weeks of randomisation. The results of the qualitative research have been published and have informed the delivery of the main trial [17, 18].

For peer review only - http://bmjopen.bmj.com/site/about/guidelines.xhtml

2.0 METHODS AND ANALYSIS

2.1 Study Design

This is a placebo controlled, double blind, pragmatic group sequential UK-wide trial to determine the effectiveness of GTN for treating retained placenta and avoiding manual removal of placenta. Women are identified as being potentially eligible for the trial if, following vaginal birth, they have a retained placenta and are at risk of needing a manual removal of placenta after failure of current management of the third stage of labour. If eligible for the trial, women are treated with either the study drug or a placebo in a double-blind fashion. Following administration of the study medication, if the placenta still remains undelivered after 15 minutes, the treating clinician determines ongoing clinical management as per standard care.

The pre-specified group sequential monitoring plan requires the Data Monitoring Committee to look at accumulating unblinded data on five occasions. The Data Monitoring Committee is using the results of these analyses to advise the Trial Steering Committee on adapting the trial design to either a) stop prematurely for futility (no prospect of establishing a treatment effect of at least 10%), or b) stop prematurely if proof beyond a reasonable doubt is established that there is a convincing treatment benefit of at least 10%. Assuming the trial recruits to its maximum size, we are aiming to recruit 1100 participants (maximum number randomised 1086, with the uplift to 1100 to account for a small allowance for withdrawal of consent or other rare loss to follow-up) over 42 months.

2.2 Study Population

2.2.1 Setting

We are recruiting women from delivery wards in UK maternity hospitals. The delivery wards are of varying size and location ensuring the results of the trial will be generalisable to the UK. At present, recruitment is being undertaken in 28 sites across the UK.

For peer review only - http://bmjopen.bmj.com/site/about/guidelines.xhtml

2.2.2 Selection of participants

As is standard practice, clinicians are undertaking the initial eligibility screening. Women are identified as being potentially eligible for the trial if, following vaginal birth, they have a retained placenta and are at risk of needing a manual removal of their placenta after failure of current management of the third stage of labour, defined as the placenta remaining undelivered after (i) 30 minutes of active management or (ii) 60 minutes of physiological management plus a further 30 minutes of active management of the third stage of labour.

2.2.3 Planned inclusion and exclusion criteria

All women will be considered for the trial who have RP after vaginal birth following failure of current management (defined as a third stage of labour lasting more than (i) 30 minutes after active management or (ii) 60 minutes following physiological followed by 30 minutes of active management. In addition, they should also satisfy the following inclusion criteria. Should a woman have any of the exclusion criteria, they will not be invited to participate in the trial.

Inclusion criteria:

Women are eligible for inclusion if they are:

- Aged ≥16 years
- Vaginal delivery (including women with a previous caesarean section and an instrumental vaginal delivery in the delivery room)
- >14 weeks gestation
- Haemodynamically stable, defined as having a heart rate ≤119 beats per minute and a systolic blood pressure of > 100mmHg

Exclusion Criteria:

Women are excluded if they have one or more of the following:

For peer review only - http://bmjopen.bmj.com/site/about/guidelines.xhtml

- Unable to give informed consent
- Suspected placenta accreta/increta/percreta
- Multiple pregnancy
- Allergy or hypersensitivity to nitrates
- Consumption of alcohol in the last 24 hours
- Concomitant use of phosphodiesterase inhibitors
- Instrumental vaginal delivery in theatre
- Contra-indications to nitrates including severe anaemia, constrictive pericarditis, extreme bradycardia, incipient glaucoma, glusose-6-phosphate dehydrogenase–deficiency, cerebral haemorrhage and brain trauma, aortic and/or mitral stenosis, angina caused by hypertrophic obstructive cardiomyopathy, circulatory collapse, cardiogenic shock, and toxic pulmonary oedema

2.3 Recruitment and Study Procedures

2.3.1 Identifying participants

Recruitment was initiated in October 2014 and is currently ongoing in the delivery wards of 28 UK maternity hospitals. Recruitment is scheduled to conclude when 1100 subjects are recruited to the trial.

We initially followed the Royal College of Obstetrics and Gynaecology best practice recommendations for recruitment and obtaining consent for intrapartum research studies [19]. These recommendations were based on the work by Vernon et al, [20] who developed a consent pathway for women with retained placenta who were recruited to the RELEASE trial of umbilical vein oxytocin for RP [21]. We refined this pathway in light of our qualitative research [17, 18] so that the consent and recruitment pathways were tailored to the specific requirements of this trial. In brief, where

For peer review only

For peer review only - http://bmjopen.bmj.com/site/about/guidelines.xhtml

possible, outline information about the trial (including researcher contact details) is made available to women antenatally, particularly those identified as being at high risk of retained placenta e.g. maternal age >30 years, pre-term birth and stillbirth. [22] More detailed information is available in antenatal clinics, on labour wards and the study website with information posters in the hospital reception areas and antenatal clinics. Following diagnosis of a retained placenta, a clinician or midwife who has received GOT-IT study training (which has sponsor approved GCP-training embedded within it), invites women to participate in the trial, provides an explanation of the study and gives potential participants an abbreviated and/or full version of the patient information sheet.

2.3.2 Informed Consent

The patient information leaflets explain that the trial is investigating whether sublingual GTN spray is useful for treating retained placenta. If a woman wishes to participate then written or verbal consent is obtained. If verbal consent is obtained, this is followed up by written consent as soon as possible in the postnatal period. Such an approach has been endorsed by the Royal College of Obstetricians and Gynaecologists in recent guidance about obtaining consent in perinatal research where consent is time critical in which it is stated that '[in acute circumstances]…provision of antenatal information to women with brief oral consent at the time of the complication is appropriate. Full written consent is then obtained at a later stage' [23]. Permission is sought to inform the woman's general practitioner that she is taking part in this trial. Due to the emergency nature of the condition (retained placenta), women are eligible to take part if they fulfil the inclusion criteria, understand what trial participation involves and give fully informed consent. A minimum time period is not feasible for trial consideration by potential participants. If a woman cannot give informed consent (e.g. due to incapacity), she is not eligible for participation.

For peer review only - http://bmjopen.bmj.com/site/about/guidelines.xhtml

For peer review only

2.3.3 Randomisation and allocation

The study drug is a metered dose sublingual spray containing 400mcg/dose of glyceryl trinitrate and a matching placebo spray. The formulation consists of glyceryl trinitrate, peppermint oil BP 88, 1,1,1,2-tetrafluroethane (HFA), and ethanol BP (96%). The placebo is identical to the active product minus the glyceryl trinitrate. Both the study drug and the matching placebo are manufactured by Pharmasol Limited, and labelled by Sharp Clinical Services (UK) Limited.

Study drugs are kept on the delivery suite and are provided to site pharmacies in pre-packed randomised permuted blocks. After consent of each participant, the investigator or trained clinical practitioner randomises eligible and consenting women to one of the two study groups by taking the next box off the shelf. After priming the pump and demonstrating its use, the clinician gives the study drug to the women to self-administer. The study is double masked so neither the participant, investigator nor trained clinical practitioner know which treatment has been allocated. The boxes are numbered and labelled so that the identity of the drug can be determined later. The number on the box is logged in the study database. Breaking of the study masking is only performed where knowledge of the treatment is absolutely necessary for safe management of the patient.

A Central Emergency unblinding interactive voice response system is operated by the Centre for Healthcare Randomised Trials (ChaRT) in Aberdeen. Only senior clinicians can unblind trial participants. The name of the clinician requesting the unblinding, the reasons for it and notification of any unblinding is sent to the Chief Investigator via e-mail. Reasons for unblinding are recorded in the participant's sources documents and in the Investigator Site Master File. Unless there is a clinical requirement, masking will not be broken until after all study data entry is complete, the validity of the data is checked, all queries resolved and the patient populations agreed.

2.3.4 Administration of study intervention

Women who have a retained placenta after vaginal birth or miscarriage following failure of management of the third stage of labour (failure of active management defined as the third stage

For peer review only - http://bmjopen.bmj.com/site/about/guidelines.xhtml

lasting more than 30 mins and failure of physiological management defined as the third stage lasting more than 60 mins, plus a further 30 minutes of active management) are considered for eligibility for the trial.

Baseline observations (maternal blood pressure (mmHg) and heart rate (beats per minute)) are taken prior to study drug administration to confirm that the woman fulfils the haemodynamic inclusion criteria (heart rate ≤119 bpm, and systolic blood pressure >100mmHg) for trial entry.

Following confirmation of eligibility, women self-administer two puffs of either GTN (800mcg) or placebo spray as a once-only, sub-lingual intervention via a pump spray. Maternal temperature and a blood sample to measure haemoglobin are measured at baseline. Heart rate, systolic blood pressure and temperature (^{o}C) are recorded at five and 15 minutes after administration of the study drug.

Sublingual GTN is maximally effective in causing uterine and cervical relaxation by five minutes post administration. Thus, if spontaneous delivery of the placenta is going to occur, we expect this to happen around five minutes after drug administration. If spontaneous placental delivery has not occurred, a further attempt to deliver the placenta by controlled cord traction is made. If the placenta remains undelivered 15 minutes after administration of the treatment we think that it is highly unlikely that it will deliver spontaneously thereafter – we consider that any further delay risks haemorrhage. Thus the decision that manual removal of placenta is needed (primary clinical outcome) is made and the participant transferred to theatre for the definitive management of manual removal of placenta as soon as possible. Additionally, if there are clinically significant side-effects (e.g. haemorrhage, symptomatic maternal hypotension and/or tachycardia) before 15 minutes have elapsed after GTN/placebo treatment, the participant is transferred to theatre for immediate manual removal of placenta.

2.3.5 Follow-Up Procedures

For peer review only - http://bmjopen.bmj.com/site/about/guidelines.xhtml

On the first post-natal day a haemoglobin sample is collected. A questionnaire relating to the woman's perception of the study drug in relation to side-effects and patient satisfaction is also completed. A second questionnaire is sent by the Central Trial Office to participants at six weeks post-deliveryThe questionnaire collects further information on patient-rated side-effects, patient rated satisfaction and health economic resource use.

Women remain in the trial unless they are unable to continue for a clinical reason or choose to withdraw consent. All other changes in status, with the exception of formal withdrawal of consent, mean the participant will be followed up for all study outcomes whenever possible.

2.4 Safety

The laws that govern reporting for Clinical Trials of Investigational Medicinal Products regulate safety reporting for the GOT-IT trial. Adverse events may occur during or after participation in the trial.

2.4.1 Definitions

An **adverse event** (AE) is any untoward medical occurrence in a clinical trial participant which does not necessarily have a causal relationship with an investigational medicinal product (IMP). Each AE is considered for severity, causality or expectedness and may be reclassified as a serious event.

An **adverse reaction** (AR) is any untoward and unintended response to the IMP which is related to any dose administered to that participant.

For peer review only - http://bmjopen.bmj.com/site/about/guidelines.xhtml

A **serious adverse event** (SAE), **serious adverse reaction** (SAR) is any AE or AR that at any dose:

- results in death of the clinical trial participant;
- is life threatening, defined as an event where the participant was at risk of death at the time of the event. It does not refer to an event which hypothetically might have caused death if it were more severe;
- requires in-patient hospitalisation or prolongation of existing hospitalisation;
- results in persistent or significant disability or incapacity;
- consists of a congenital anomaly or birth defect;
- results in any other significant medical event not meeting the criteria above.

A suspected unexpected serious adverse reaction (SUSAR) is any AR that is classified as serious and is suspected to be caused by the IMP, that it is not consistent with the information about the IMP in the summary of the medicinal product characteristics (SmPC).

2.4.2 Identifying AEs and SAEs

All AEs and SAEs are recorded from the time a participant signs the consent form to take part in the study until the six week postnatal outcome assessment point.

Participants are asked about the occurrence of AEs/SAEs prior to discharge from the hospital and in the six week postnatal questionnaire. Open-ended and non-leading verbal questioning of the participant are used to enquire about AE/SAE occurrence at discharge. The six week postnatal questionnaire also asks participants if they have seen their general practitioner, been admitted to hospital, or been prescribed any medication. If there is any doubt as to whether a clinical observation is an AE, the event is recorded. AEs and SAEs are also identified via information from support departments e.g. laboratories.

For peer review only

2.4.3 Recording AEs and SAEs

When an AE/SAE occurs, it is the responsibility of the Principal Investigator to review all documentation (e.g. hospital notes, laboratory and diagnostic reports) related to the event. The Principal Investigator records all relevant information on the SAE form (if the AE meets the criteria of SAE). Information to be collected includes type of event, onset date, Investigator assessment of severity and causality, date of resolution as well as treatment required, investigations needed and outcome.

The clinician will assess all reported SAEs. The events detailed in the list below are 'expected' and will not be reported to the Co-Sponsors as an SAE but will be recorded in the case report form as a hospitalisation or outcome and presented to the Data Monitoring Committee, as part of the ongoing safety review.

In this trial, the following events are not considered SAEs:

i) Pregnancy is not considered an AE or SAE, as it is part of the inclusion criteria.

ii) Hospitalisations for treatment planned prior to randomisation and hospitalisation for elective treatment of a pre-existing condition will not be considered as an SAE. This includes pregnancy.

iii) Fall in haemoglobin of more than 15% between recruitment and the first postnatal day

iv) Manual removal of placenta in theatre.

v) Need for earlier than planned manual removal of placenta on the basis of the clinical condition.

For peer review only

375 vi) Fall in systolic or diastolic blood pressure of more than 15mmHg and/or increase in pulse
376 of more than 20 beats/minute between baseline and five and 15 minutes post-
377 administration of active/placebo treatment.

378 vii) Need for blood transfusion between time of delivery and discharge from hospital.

379 viii) Need for general anaesthesia.

380 ix) Maternal pyrexia (one or more temperature reading of more than 38^{0}C within 72 hours of
381 delivery or discharge from hospital if discharge occurs sooner).

382 x) Sustained uterine relaxation after removal of placenta requiring uterotonics.

383

384 **2.4.4 Assessment of AEs and SAEs**

385 Seriousness, causality, severity and expectedness of each AE are assessed by the Principal
386 Investigator. AEs are assessed as though the participant is taking active IMP. Cases that are
387 considered serious, possibly, probably or definitely related to IMP and unexpected (i.e. SUSARs) are
388 unblinded. The Chief Investigator may not downgrade an event that has been assessed by an
389 Investigator as an SAE or SUSAR, but can upgrade an AE to an SAE, SAR or SUSAR if appropriate.

390 **Assessment of Seriousness**

391 The Investigator makes an assessment of seriousness as defined in 2.4.1.

392 **Assessment of Causality**

393 The Investigator makes an assessment of whether the AE/SAE is likely to be related to the IMP
394 according to the definitions below.

395 • Unrelated: where an event is not considered to be related to the IMP.
396 • Possibly Related: The nature of the event, the underlying medical condition, concomitant
397 medication or temporal relationship make it possible that the AE has a causal relationship to

For peer review only

the study drug. The assessment of causality is made against the reference safety information found in the SmPC.

Where non Investigational Medicinal Products (NIMPs) e.g. rescue/escape drugs are given: if the AE is considered to be related to an interaction between the IMP and the NIMP, or where the AE might be linked to either the IMP or the NIMP but cannot be clearly attributed to either one of these, the event will be considered as an AR. Alternative causes such as natural history of the underlying disease, other risk factors and the temporal relationship of the event to the treatment are considered and investigated, however the blind will not be broken for the purpose of making this assessment.

Assessment of Expectedness

If an event is judged to be an AR, the evaluation of expectedness will be made based on knowledge of the reaction and the relevant product information documented in the SmPC. Events are classed as either expected: (the AR is consistent with the toxicity of the IMP listed in the SmPC) or Unexpected: (the AR is not consistent with the toxicity in the SmPC).

Assessment of Severity

The Investigator makes an assessment of severity for each AE/SAE and records this on the SAE form according to one of the following categories:

- Mild: an event that is easily tolerated by the participant, causing minimal discomfort and not interfering with every day activities.
- Moderate: an event that is sufficiently discomforting to interfere with normal everyday activities.
- Severe: an event that prevents normal everyday activities.

2.4.5 Reporting of SAEs/SAR/SUSARs

Once the Investigator becomes aware that an SAE has occurred in a study participant, the information is reported to the Academic and Clinical Central Office for Research and Development (ACCORD) Research Governance and Quality Assurance Office (Edinburgh University and NHS Lothian).

For peer review only - http://bmjopen.bmj.com/site/about/guidelines.xhtml

immediately or within 24 hours. If the Investigator does not have all information regarding an SAE, they should not wait for this additional information before notifying ACCORD. The SAE report form can be updated when the additional information is received.

The SAE report provides an assessment of causality and expectedness at the time of the initial report to ACCORD. Where missing information has not been sent to ACCORD after an initial report, ACCORD contacts the Investigator and requests the missing information until this is supplied.

All reports sent to ACCORD and any follow up information are retained by the Investigator in the Investigator Site File. ACCORD also inform the trial office who check that the SAE is added to the CHaRT database.

2.4.6 Regulatory reporting requirements

The ACCORD Research Governance & QA Office is responsible for pharmacovigilance reporting on behalf of the co-sponsors (Edinburgh University and NHS Lothian). The ACCORD Research Governance and Quality Assurance Office has a legal responsibility to notify the regulatory competent authority and the Research Ethics Committee that approved the trial. Fatal or life threatening SUSARs are reported no later than seven calendar days and all other SUSARs are reported no later than 15 calendar days after ACCORD is first aware of the reaction. ACCORD informs Investigators at participating sites of all SUSARs and any other arising safety information. An Annual Safety Report/Development Safety Update Report is submitted, by ACCORD, to the regulatory authorities and Research Ethics Committees listing all SARs and SUSARs.

2.4.7 Follow up procedures

After initially recording an AE or recording and reporting an SAE, the Investigator follows each participant until resolution or death of the participant. Follow up information on an SAE is reported

For peer review only - http://bmjopen.bmj.com/site/about/guidelines.xhtml

to the ACCORD office. AEs still present in participants at the six week postnatal outcome assessment point are monitored until resolution of the event or until no longer medically indicated.

2.5 Outcome Measures

The GOT-IT trial is assessing whether sublingual GTN spray is clinically and cost effective in treating retained placenta after vaginal delivery, reducing the need for manual removal of placenta following failure of current management.

2.5.1 Primary outcome measures

The primary outcome is measured over four interrelated domains of clinical, safety, patient-sided and economic.

The primary clinical outcome is need for manual removal of placenta, defined as the placenta remaining undelivered 15 minutes post study treatment and/or being required within 15 minutes of treatment due to safety concerns.

The primary safety outcome is measured blood loss between administration treatment and transfer to the postnatal ward or other clinical area.

The primary patient-sided outcome is satisfaction with treatment, a side effect profile assessed by a questionnaire administered at discharge from hospital and a postal questionnaire sent to the participant six weeks post hospital discharge.

The primary economic outcome is net incremental costs (or cost savings) to the National Health Service of using GTN versus standard practice. Costs include GTN (dose and time to administer drug), monitoring of the woman and delivering the placenta if effective), manual removal of placenta if required, and further health service resource use to six weeks post discharge.

For peer review only - http://bmjopen.bmj.com/site/about/guidelines.xhtml

2.5.2 Secondary Outcomes

The secondary endpoints are measured over two interrelated domains of clinical and economic outcomes. There are no secondary patient-sided or safety outcomes.

The secondary clinical endpoints include: i) Fall in haemoglobin of more than 15% between recruitment and the first postnatal day; ii) time from randomisation to delivery of placenta; iii) manual removal of placenta in theatre; iv) need for earlier than planned manual removal of placenta on the basis of the clinical condition, v) fall in systolic or diastolic blood pressure of more than 15mm Hg and/or increase in pulse of more than 20 beats/minute between baseline and five and 15 minutes post-administration of active/placebo treatment; vi) need for blood transfusion between time of delivery and discharge from hospital; vii) need for general anaesthesia; viii) maternal pyrexia (one or more temperature reading of more than 38°C within 72 hours of delivery or discharge from hospital if discharge occurs sooner); ix) sustained uterine relaxation after removal placenta requiring uterotonics; x) readmission to hospital for any reason within six weeks postpartum.

Estimated health service costs will be compared with multiple trial outcomes in a cost-consequence analysis of GTN versus standard practice. The costs and consequences of the alternative approaches to treatment will be summarised using a balance sheet, highlighting the costs and outcomes favouring each treatment option.

2.6 Data Collection and Processing

2.6.1 Collection of Outcome Data

The primary clinical outcome (need for manual removal of placenta, defined as the placenta remaining undelivered 15 minutes post treatment and/or being required within 15 minutes of treatment due to safety concerns) is collected 15 minutes post administration of the study drug. The primary safety outcome (measured blood loss between administration treatment and transfer to the postnatal ward/other clinical area) is recorded immediately prior to transfer from theatre to the

For peer review only - http://bmjopen.bmj.com/site/about/guidelines.xhtml

postnatal ward or other clinical area (e.g. labour ward high dependency). The primary patient satisfaction outcome is collected at six weeks by a self-completed satisfaction questionnaire and the primary economic outcome will be computed from costs incurred at initial management of retained placenta and management of further complications and self-reported health service usage and side-effects recorded from the postnatal patient questionnaires.

Outcome data are collected on an electronic case report form and paper-based questionnaires by research midwives and nurses who have been specifically trained in data collection for the trial. The electronic case record form comprises the following sections: baseline data, clinical observations and placenta delivery, first post natal day, discharge, six week post natal check, safety events, discharge and follow-up questionnaires. Health service use in the six weeks following discharge is collected via a small number of resource use questions included in the postnatal questionnaires. Resource use will be evaluated using routine sources of nationally relevant unit costs. Women are permitted to receive all standard care medical treatments for postpartum complications including any given for medical management of retained placenta. Concomitant medications are recorded from 24 hours prior to signing of the consent forms until 24 hours after administration of the study drug. Analgesic or antibiotic medications are recorded from 24 hours prior to signing of informed consent until six weeks after administration of study drug.

2.7 Sample Size, proposed recruitment rate and milestones

2.7.1 Sample size

The trial uses a group sequential design as opposed to a fixed sample design to allow for the uncertainty of two critical factors. First, there is considerable uncertainty about how many women who are eligible for the trial will actually go on and require surgery due to a) lack of knowledge of the frequency of spontaneous delivery of the placenta beyond the time frame that GTN is known to have a pharmacological effect, and b) the effect of variations in local clinical practice in relation to organising manual removal of placentas. Secondly, the magnitude of the benefit from GTN spray is

For peer review only

For peer review only - http://bmjopen.bmj.com/site/about/guidelines.xhtml

unknown. To properly reflect these uncertainties, we are using a group sequential design. This design maximises our chances of efficiently detecting and estimating the true benefit of treatment in the quickest time with the right number of participants, and gives us the opportunity to abandon the trial if it turns out that no worthwhile treatment effect exists (via futility analyses).

In discussion with clinicians and women, a minimally clinically important difference of an absolute reduction of 10% in the need for manual removal of placenta would need to be observed in order to accommodate this intervention (sublingual GTN) in clinical practice. Given that the maximum variability in a binary outcome (yes or no) occurs at a 50% rate in the placebo spray arm, a fixed sample approach would need 1038 women (519 in each group) to demonstrate a 10% change from 50% on placebo to 40% on GTN spray. As the outcome (surgery occurred – yes or no) takes place 15 minutes after the intervention has been administered there will be minimal loss to follow-up and therefore, no requirement for a trial much larger than this. Using a group sequential design incorporating a Lan-DeMets alpha spending function with O'Brien-Fleming boundaries, our trial has a maximum sample size of 1086 (with a small inflation to 1100 to allow for withdrawal of consent of other rare loss to follow-up). This allows for five planned interim evaluations by the Data Monitoring Committee of accruing data at 218, 436, 652 and 870 participants with a final look (if the trial proceeds to full size) at 1086 women.

2.8 Analyses Plan

2.8.1 Statistical Analysis

The statistical analyses will be according to the Intention-To-Treat Principle and will be governed by a Statistical Analysis Plan which will govern all statistical aspects of the study, including the statistical methods for the analysis of all the outcomes, approaches to missing data, and sensitivity type analyses to investigate the robustness of any findings reported. The Statistical Analysis Plan will be finalised (including the decision to undertake pre-specified subgroup analyses or not) prior to the

For peer review only - http://bmjopen.bmj.com/site/about/guidelines.xhtml

data being locked for final analysis. The interim analyses will be specified within the Data Monitoring Committee Charter and the result of the interim analyses will be in strict confidence and unknown to the trial research group apart from the study statistician. The primary outcomes: clinical (manual removal of placenta), safety (blood loss), patient satisfaction and economic (NHS costs) will be analysed using generalised linear models appropriate to the distribution of each outcome. These models will adjust for relevant baseline factors, and sensitivity analyses will assess the robustness of the findings to any missing data (which is expected to be very low as the primary outcome is measured within minutes of receiving the intervention). The interim analysis will be performed in East [24] and all remaining analysis will be done in Stata [25]. Significance will be defined as $P<0.05$. In the event pre-specified formal subgroup analyses of the primary outcome are undertaken, we will formally assess subgroup(s) by fitting a subgroup * randomized group interaction term and these interactions will be assessed against a stricter level of significance of $P<0.01$ to guard against chance findings.

2.8.2 Economic Evaluation

While the cost of GTN is low, there will still be costs associated with its administration and the monitoring and management of women thereafter. Should the intervention prove effective, it will be important in the context of scarce maternity resources to explicitly quantify the net costs (or cost savings) associated with its use. We have therefore made provision to carry out a simple cost analysis using clinical and resource use data, which is being collected for individual participants recruited to the trial. This analysis will explicitly quantify the difference in mean costs between the active intervention and placebo arm. Research costs associated with placebo delivery will be factored out of the analysis to estimate the incremental cost (or cost savings) of the active intervention versus standard practice.

For peer review only - http://bmjopen.bmj.com/site/about/guidelines.xhtml

Resource use associated with the alternative management strategies will be estimated from the time of randomisation through to six weeks post-partum. This will include: 1) staff time for administering the drug to patients; 2) resource use associated with any complications arising following administration of the study drug (e.g. blood pressure and/or heart rate monitoring); 3) subsequent costs associated with delivery of the placenta (either spontaneously or operatively); and 4) subsequent health service contact relating to retained products of conception up to six weeks post discharge. Resource use collected for individual participants will be valued using accepted national unit costs for drugs, staff time, MROP and subsequent health care contacts [26-28]. The total cost of care for each participant will be calculated for each individual.

The mean costs will be summarised by treatment allocation group, and the incremental cost (or cost saving) associated with the use of GTN will be estimated using an appropriately specified general linear model. The cost data will be presented alongside the primary and secondary outcome data in a cost-consequence balance sheet, indicating which strategy each outcome favours [29].

2.9 Organisation: Trial Management, Oversight and Governance Arrangements

2.9.1 Trial management

The trial is being jointly run by CHaRT based at the University of Aberdeen, the Chief Investigator and the ACCORD office at the University of Edinburgh. The trial management and governance are as recommended by The Research Governance Framework and The Medicines for Human Use (Clinical Trials) Regulations 2004. Briefly, we have set up a Trial Steering Committee and a Data Monitoring Committee led by individuals independent of the project and the institutions involved. The membership of the Trial Steering Committee includes independent members including a lay representative. A separate and independent Data Monitoring Committee has also been convened. The

For peer review only - http://bmjopen.bmj.com/site/about/guidelines.xhtml

Data Monitoring Committee met during trial set-up to agree terms of reference and is meeting a further five times during recruitment to monitor accumulating data and oversee safety issues. The Data Monitoring Committee will meet one final time after unblinding to assess overall data validity and analyses. The Trial Steering Committee is meeting approximately seven times with the meeting spacing being matched to that of the Data Monitoring Committee. Finally, we have regular Project Management Group meetings and Research Midwives Meetings to ensure consistency of trial processes across sites and share best practice for recruitment, consent and data collection.

2.9.2 Research governance and compliance

2.9.2.1 Research Governance

ACCORD is providing oversight that the appropriate research and competent authority approvals are in place, pharmacovigilance services and is ensuring the trial is conducted in accordance with the principles of the International Conference on Harmonisation Tripartite Guideline for Good Clinical Practice.

2.9.2.2 Data protection

The trial complies with the Data Protection Act 1998 and regular monitoring and checks are in place to ensure compliance. Data is being stored in accordance with the Act and will be archived to a secure data storage facility. The consent form states that other researchers may wish to access (anonymised) data in the future. The Senior Information Technology Manager (in collaboration with the trial statistician and trial manager) will manage access rights to the data set. Prospective new users must demonstrate compliance with data protection, legal and ethical guidelines before any data are released. We anticipate that anonymised trial data will be shared with other researchers to enable international prospective meta-analyses.

For peer review only - http://bmjopen.bmj.com/site/about/guidelines.xhtml

2.9.2.3 Sponsorship

The University of Edinburgh and NHS Lothian are the co-sponsors for the trial. Co-sponsorship responsibilities are detailed in an agreement. Responsibilities sub-contracted to CHaRT are also detailed in an agreement which detail the responsibilities surrounding centralised trial administration, database support and economic and statistical analyses.

2.9.2.3 Quality Assurance

An independent risk assessment has been performed to determine the level of monitoring required and if an audit should be performed before/during/after the study and if so, at what locations and at what frequency.

2.9.2.4 Data Handling, Record Keeping and Archiving

The local principal investigator or research midwives enter locally-collected data from the study centres into the web-based data collection application. The 6-week follow-up questionnaires are sent from and returned to the Study Office in Edinburgh and are inputted by the staff in the Study Office in Edinburgh. CHaRT is working with the Study Office in Edinburgh and with local Research Midwives to ensure that the data is as complete and accurate as possible.

3.0 Ethics, Regulatory Approvals and Dissemination

3.1 Ethics and Regulatory Approvals

The Trial is registered on the International Standard Randomised Controlled Trial Number (ISRCTN) as ISCRTN88609453. Ethical approval has been obtained from the North East- Newcastle & North Tyneside 2 Research Ethics Committee [13/NE/0339]. Approval has also been obtained from the

For peer review only - http://bmjopen.bmj.com/site/about/guidelines.xhtml

Medicines and Healthcare Products Regulatory Authority (MHRA) [2013-003819-42] and the Health Research Authority as well as approvals from the local Trust Research and Development Offices. The study has been adopted to the National Institute for Health Research Clinical Research Network (NIHR CRN) Portfolio [http://www.nihr.ac.uk/research-and-impact/nihr-clinical-research-network-portfolio/]. The necessary trial insurance is provided by the University of Edinburgh.

3.2 Dissemination

The dissemination plans include the Health Technology Assessment monograph, presentation at international scientific meetings, and publication of the results of the main trial and each of the secondary endpoints in high impact peer reviewed journals. If all grant-holders and researcher staff fulfil authorship rules, group authorship will be used under the collective title of 'the GOT-IT Trial Group'. The Chief Investigator, Trial Manager, and possibly other members of the trial group will take responsibility for drafting the paper and this will be recognised by line 'the Chief Investigator (as primary author), followed by the other authors and the GOT-IT Trial Group'. Authorship of the qualitative sub-study publications [17, 18] has been attributed to Chief Investigator and the named individual(s). To maintain interest in the study, we are also publishing GOT-IT newsletters at intervals to trial staff and collaborators.

For peer review only

For peer review only - http://bmjopen.bmj.com/site/about/guidelines.xhtml

665 **A. Contributorship statement**

666 FCD conceived and designed the study. FCD, JN, JL, GS, GM, AM, JB, MP, CC, SBS, NH and JEN drafted the original grant proposal and trial protocol. JN and JH provide methodological and statistical expertise. JL and NH provide expertise in the qualitative studies. FCD, JEN and SBS provide expertise in the clinical outcomes. KFC and SM have drafted protocol revisions and have both been trial managers for the trial. MF and AM provide database and trial support from the trials unit. JB and CC provide lay input. FCD and KFC drafted the manuscript. KFC has responsibilities for day-to-day running of the trial including participant recruitment, data collection and liaising with other sites. All authors critically reviewed and approved the final version of the manuscript.

674 **B. Competing interests statement**

675 The authors have no competing interests to declare.

677 **C. Funding statement**

678 The GOT-IT Trial (trial registration number: ISCRTN 88609453) is funded by the National Institute for Health Research Health Technology Assessment (HTA) programme (Project number 12/29/01). The views and opinions expressed therein are those of the authors and do not necessarily reflect those of the HTA, the National Institute for Health Research, the NHS or the Department of Health. This work was undertaken in the MRC Centre for Reproductive Health which is funded by MRC Centre grant (MRC G1002033).

684 **D. Data sharing statement**

685 Not applicable. This article is a pre-publication protocol for the GOT-IT trial and contains no data.

For peer review only

4.0 References:

1. NICE, *NICE clinical guideline 55: Intrapartum care: care of healthy women and their babies during childbirth.* 2012.
2. Stones, R.W., C.M. Paterson, and N.J. Saunders, *Risk factors for major obstetric haemorrhage.* Eur J Obstet Gynecol Reprod Biol, 1993. **48**(1): p. 15-8.
3. Combs, C.A. and R.K. Laros, Jr., *Prolonged third stage of labor: morbidity and risk factors.* Obstet Gynecol, 1991. **77**(6): p. 863-7.
4. Cheung, W.M., et al., *The retained placenta: historical and geographical rate variations.* J Obstet Gynaecol, 2011. **31**(1): p. 37-42.
5. Ely, J.W., et al., *The association between manual removal of the placenta and postpartum endometritis following vaginal delivery.* Obstet Gynecol, 1995. **86**(6): p. 1002-6.
6. Combs, C.A., E.L. Murphy, and R.K. Laros, Jr., *Factors associated with postpartum hemorrhage with vaginal birth.* Obstet Gynecol, 1991. **77**(1): p. 69-76.
7. Abdel-Aleem, H., M.A. Abdel-Aleem, and O.M. Shaaban, *Nitroglycerin for management of retained placenta. .* Cochrane Database of Systematic Reviews, 2015(11).
8. Barnes, F., *Hour-Glass Contraction of the Uterus Treated with Nitrite of Amyl.* Br Med J, 1882. **1**(1107): p. 377.
9. Farley, A.E., C.H. Graham, and G.N. Smith, *Contractile properties of human placental anchoring villi.* Am J Physiol Regul Integr Comp Physiol, 2004. **287**(3): p. R680-5.
10. Weeks, A.D., *The retained placenta.* Best Pract Res Clin Obstet Gynaecol, 2008. **22**(6): p. 1103-17.
11. Bullarbo, M., J. Tjugum, and E. Ekerhovd, *Sublingual nitroglycerin for management of retained placenta.* Int J Gynaecol Obstet, 2005. **91**(3): p. 228-32.
12. Redick, L.F. and E. Livingston, *A new preparation of nitroglycerin for uterine relaxation.* Int J Obstet Anesth, 1995. **4**(1): p. 14-6.
13. Laslett, L.J. and L. Baker, *Sublingual nitroglycerin administered by spray versus tablet: comparative timing of hemodynamic effects.* Cardiology, 1990. **77**(4): p. 303-10.
14. Marmor, A., *Comparative evaluation of a new formulation of isosorbide dinitrate oral spray and sublingual nitroglycerin tablets.* Am J Cardiol, 1990. **65**(21): p. 43J-45J.
15. Curry, S.H., et al., *Plasma concentrations and hemodynamic effects of intravenous, sublingual, and aerosolized nitroglycerin in patients undergoing cardiac catheterization.* Biopharm Drug Dispos, 1993. **14**(2): p. 107-18.
16. Wight, L.J., et al., *A large scale comparative study in general practice with nitroglycerin spray and tablet formulations in elderly patients with angina pectoris.* Eur J Clin Pharmacol, 1992. **42**(3): p. 341-2.
17. Hallowell, N., et al., *The role of therapeutic optimism in recruitment to a clinical trial in a peripartum setting: balancing hope and uncertainty.* Trials, 2016. **17**(1): p. 267.
18. Lawton, J., et al., *Recruiting and consenting into a peripartum trial in an emergency setting: a qualitative study of the experiences and views of women and healthcare professionals.* Trials, 2016. **17**(1): p. 195.
19. RCOG, *Obtaining valid consent to participate in research while in labour*, in *Clinical Governance Advice No.6a*2010, RCOG: London.
20. Vernon, G., Z. Alfirevic, and A. Weeks, *Issues of informed consent for intrapartum trials: a suggested consent pathway from the experience of the Release trial [ISRCTN13204258].* Trials, 2006. **7**: p. 13.
21. Weeks, A.D., et al., *Umbilical vein oxytocin for the treatment of retained placenta (Release Study): a double-blind, randomised controlled trial.* Lancet, 2010. **375**(9709): p. 141-7.
22. Coviello, E.M., et al., *Risk factors for retained placenta.* Am J Obstet Gynecol, 2015. **213**(6): p. 864 e1-864 e11.
23. RCOG, *Obtaining Valid Consent to Participate in Perinatal Research Where Consent is Time Critical*, in *Clinical Governance Advice no. 6a*, ROCG, Editor 2016, RCOG: London.

For peer review only

For peer review only - http://bmjopen.bmj.com/site/about/guidelines.xhtml

24. 6, E., *Statistical software for the design, simulation and monitoring clinical trials*, 2016, Cytel Inc., Cambridge MA.
25. StataCorp., *Stata Statistical Software: Release 14.* , 2015, College Station, TX: StataCorp LP.
26. Complete, B.N.F.M., 2017.
27. Curtis, L., *Unit Costs of Health and Social Care 2016*, 2016, Canterbury: Personal Social Services Research Unit.
28. Health, D.o., *NHS reference costs 2015-2016* 2015: London: UK.
29. NICE., *Approaches to bespoke economic evaluation* in *Developing NICE guidelines: the manual.*, NICE, Editor. 2014.

For peer review only

SPIRIT

STANDARD PROTOCOL ITEMS: RECOMMENDATIONS FOR INTERVENTIONAL TRIALS

SPIRIT 2013 Checklist: Recommended items to address in a clinical trial protocol and related documents*

Section/item	Item No	Description	Addressed on page number
**Administrative information**			
Title	1	Descriptive title identifying the study design, population, interventions, and, if applicable, trial acronym	Page 1
Trial registration	2a	Trial identifier and registry name. If not yet registered, name of intended registry	Page 3
	2b	All items from the World Health Organization Trial Registration Data Set	/
Protocol version	3	Date and version identifier	Page
Funding	4	Sources and types of financial, material, and other support	Page 3
Roles and responsibilities	5a	Names, affiliations, and roles of protocol contributors	Page 1
	5b	Name and contact information for the trial sponsor	Page 17
	5c	Role of study sponsor and funders, if any, in study design; collection, management, analysis, and interpretation of data; writing of the report; and the decision to submit the report for publication, including whether they will have ultimate authority over any of these activities	Page 17
	5d	Composition, roles, and responsibilities of the coordinating centre, steering committee, endpoint adjudication committee, data management team, and other individuals or groups overseeing the trial, if applicable (see Item 21a for data monitoring committee)	Page 23

For peer review only

**Introduction**			
Background and rationale	6a	Description of research question and justification for undertaking the trial, including summary of relevant studies (published and unpublished) examining benefits and harms for each intervention	Pages 4 & 5
	6b	Explanation for choice of comparators	Pages 4 & 5
Objectives	7	Specific objectives or hypotheses	Pages 5 & 6
Trial design	8	Description of trial design including type of trial (eg, parallel group, crossover, factorial, single group), allocation ratio, and framework (eg, superiority, equivalence, noninferiority, exploratory)	Pages 5 & 6
**Methods: Participants, interventions, and outcomes**			
Study setting	9	Description of study settings (eg, community clinic, academic hospital) and list of countries where data will be collected. Reference to where list of study sites can be obtained	Pages 7 & 8
Eligibility criteria	10	Inclusion and exclusion criteria for participants. If applicable, eligibility criteria for study centres and individuals who will perform the interventions (eg, surgeons, psychotherapists)	Pages 8 & 9
Interventions	11a	Interventions for each group with sufficient detail to allow replication, including how and when they will be administered	Pages 10 & 11
	11b	Criteria for discontinuing or modifying allocated interventions for a given trial participant (eg, drug dose change in response to harms, participant request, or improving/worsening disease)	Page 11
	11c	Strategies to improve adherence to intervention protocols, and any procedures for monitoring adherence (eg, drug tablet return, laboratory tests)	Pages 11
	11d	Relevant concomitant care and interventions that are permitted or prohibited during the trial	Page 11
Outcomes	12	Primary, secondary, and other outcomes, including the specific measurement variable (eg, systolic blood pressure), analysis metric (eg, change from baseline, final value, time to event), method of aggregation (eg, median, proportion), and time point for each outcome. Explanation of the clinical relevance of chosen efficacy and harm outcomes is strongly recommended	Page 11, 18 - 20
Participant timeline	13	Time schedule of enrolment, interventions (including any run-ins and washouts), assessments, and visits for participants. A schematic diagram is highly recommended (see Figure)	Page 11, 18 - 20

For peer review only - http://bmjopen.bmj.com/site/about/guidelines.xhtml

Sample size	14	Estimated number of participants needed to achieve study objectives and how it was determined, including clinical and statistical assumptions supporting any sample size calculations	Pages 20 & 21
Recruitment	15	Strategies for achieving adequate participant enrolment to reach target sample size	Pages 20 & 21
**Methods: Assignment of interventions (for controlled trials)**			
Allocation:			
Sequence generation	16a	Method of generating the allocation sequence (eg, computer-generated random numbers), and list of any factors for stratification. To reduce predictability of a random sequence, details of any planned restriction (eg, blocking) should be provided in a separate document that is unavailable to those who enrol participants or assign interventions	Pages 10 & 11
Allocation concealment mechanism	16b	Mechanism of implementing the allocation sequence (eg, central telephone; sequentially numbered, opaque, sealed envelopes), describing any steps to conceal the sequence until interventions are assigned	Pages 10 & 11
Implementation	16c	Who will generate the allocation sequence, who will enrol participants, and who will assign participants to interventions	Pages 10 & 11
Blinding (masking)	17a	Who will be blinded after assignment to interventions (eg, trial participants, care providers, outcome assessors, data analysts), and how	Pages 10 & 11
	17b	If blinded, circumstances under which unblinding is permissible, and procedure for revealing a participant's allocated intervention during the trial	Pages 10 & 11
**Methods: Data collection, management, and analysis**			
Data collection methods	18a	Plans for assessment and collection of outcome, baseline, and other trial data, including any related processes to promote data quality (eg, duplicate measurements, training of assessors) and a description of study instruments (eg, questionnaires, laboratory tests) along with their reliability and validity, if known. Reference to where data collection forms can be found, if not in the protocol	Pages 18 - 20
	18b	Plans to promote participant retention and complete follow-up, including list of any outcome data to be collected for participants who discontinue or deviate from intervention protocols	Pages 18 - 20

Data management	19	Plans for data entry, coding, security, and storage, including any related processes to promote data quality (eg, double data entry; range checks for data values). Reference to where details of data management procedures can be found, if not in the protocol	Pages 18 - 20
Statistical methods	20a	Statistical methods for analysing primary and secondary outcomes. Reference to where other details of the statistical analysis plan can be found, if not in the protocol	Pages 20 - 22
	20b	Methods for any additional analyses (eg, subgroup and adjusted analyses)	Pages 20 - 22
	20c	Definition of analysis population relating to protocol non-adherence (eg, as randomised analysis), and any statistical methods to handle missing data (eg, multiple imputation)	Pages 20 - 23
**Methods: Monitoring**			
Data monitoring	21a	Composition of data monitoring committee (DMC); summary of its role and reporting structure; statement of whether it is independent from the sponsor and competing interests; and reference to where further details about its charter can be found, if not in the protocol. Alternatively, an explanation of why a DMC is not needed	Pages 24 - 25
	21b	Description of any interim analyses and stopping guidelines, including who will have access to these interim results and make the final decision to terminate the trial	Pages 20 - 25
Harms	22	Plans for collecting, assessing, reporting, and managing solicited and spontaneously reported adverse events and other unintended effects of trial interventions or trial conduct	Pages 20 - 25
Auditing	23	Frequency and procedures for auditing trial conduct, if any, and whether the process will be independent from investigators and the sponsor	Pages 20 - 25
**Ethics and dissemination**			
Research ethics approval	24	Plans for seeking research ethics committee/institutional review board (REC/IRB) approval	Page 25 - 26
Protocol amendments	25	Plans for communicating important protocol modifications (eg, changes to eligibility criteria, outcomes, analyses) to relevant parties (eg, investigators, REC/IRBs, trial participants, trial registries, journals, regulators)	Page 25 - 26

For peer review only - http://bmjopen.bmj.com/site/about/guidelines.xhtml

Consent or assent	26a	Who will obtain informed consent or assent from potential trial participants or authorised surrogates, and how (see Item 32)	Page 10
	26b	Additional consent provisions for collection and use of participant data and biological specimens in ancillary studies, if applicable	Not applicable
Confidentiality	27	How personal information about potential and enrolled participants will be collected, shared, and maintained in order to protect confidentiality before, during, and after the trial	Page 24
Declaration of interests	28	Financial and other competing interests for principal investigators for the overall trial and each study site	Page 3
Access to data	29	Statement of who will have access to the final trial dataset, and disclosure of contractual agreements that limit such access for investigators	Page 24 & 25
Ancillary and post-trial care	30	Provisions, if any, for ancillary and post-trial care, and for compensation to those who suffer harm from trial participation	Page 25
Dissemination policy	31a	Plans for investigators and sponsor to communicate trial results to participants, healthcare professionals, the public, and other relevant groups (eg, via publication, reporting in results databases, or other data sharing arrangements), including any publication restrictions	Page 25 and 26
	31b	Authorship eligibility guidelines and any intended use of professional writers	Page 25 and 26
	31c	Plans, if any, for granting public access to the full protocol, participant-level dataset, and statistical code	Not applicable
**Appendices**			
Informed consent materials	32	Model consent form and other related documentation given to participants and authorised surrogates	If requested
Biological specimens	33	Plans for collection, laboratory evaluation, and storage of biological specimens for genetic or molecular analysis in the current trial and for future use in ancillary studies, if applicable	Not applicable

*It is strongly recommended that this checklist be read in conjunction with the SPIRIT 2013 Explanation & Elaboration for important clarification on the items. Amendments to the protocol should be tracked and dated. The SPIRIT checklist is copyrighted by the SPIRIT Group under the Creative Commons "Attribution-NonCommercial-NoDerivs 3.0 Unported" license.

For peer review only - http://bmjopen.bmj.com/site/about/guidelines.xhtml
